# Genetics of Charcot-Marie-Tooth (CMT) Disease within the Frame of the Human Genome Project Success

**DOI:** 10.3390/genes5010013

**Published:** 2014-01-22

**Authors:** Vincent Timmerman, Alleene V. Strickland, Stephan Züchner

**Affiliations:** 1 Peripheral Neuropathy Group, Molecular Genetics Department, VIB, University of Antwerp, Universiteitsplein 1, Antwerpen B2610, Belgium; 2 Neurogenetics Group, Institute Born Bunge, University of Antwerp, Antwerpen B2610, Belgium; 3 Department of Human Genetics, Hussman Institute for Human Genomics, University of Miami Miller School of Medicine, Biomedical Research Building, Room 523, LC: M-860, 1501 NW 10 Ave., Miami, FL 33136, USA; E-Mail: AStrickland@med.miami.edu

**Keywords:** Charcot-Marie-Tooth, peripheral neuropathy, genomic disorders, gene discoveries, next generation sequencing

## Abstract

Charcot-Marie-Tooth (CMT) neuropathies comprise a group of monogenic disorders affecting the peripheral nervous system. CMT is characterized by a clinically and genetically heterogeneous group of neuropathies, involving all types of Mendelian inheritance patterns. Over 1,000 different mutations have been discovered in 80 disease-associated genes. Genetic research of CMT has pioneered the discovery of genomic disorders and aided in understanding the effects of copy number variation and the mechanisms of genomic rearrangements. CMT genetic study also unraveled common pathomechanisms for peripheral nerve degeneration, elucidated gene networks, and initiated the development of therapeutic approaches. The reference genome, which became available thanks to the Human Genome Project, and the development of next generation sequencing tools, considerably accelerated gene and mutation discoveries. In fact, the first clinical whole genome sequence was reported in a patient with CMT. Here we review the history of CMT gene discoveries, starting with technologies from the early days in human genetics through the high-throughput application of modern DNA analyses. We highlight the most relevant examples of CMT genes and mutation mechanisms, some of which provide promising treatment strategies. Finally, we propose future initiatives to accelerate diagnosis of CMT patients through new ways of sharing large datasets and genetic variants, and at ever diminishing costs.

## 1. Introduction

Charcot-Marie-Tooth (CMT) disease was so named to acknowledge J.M. Charcot, P. Marie, and H.H. Tooth, who originally described this inherited peripheral neuropathy in the 19th century [1,2]. CMT occurs worldwide with an estimated prevalence of 1/2,500. CMT is a neuromuscular disorder characterized by progressive and length-dependent degeneration of peripheral nerves resulting in muscle weakness and wasting in distal limbs, feet and hands. Onset varies from childhood to late adulthood and clinical severity ranges from mild to severe between patients. The neurophysiological and neuropathological defects in the motor and/or sensory nerves create foot deformities, walking disabilities, wheelchair dependence, and sensory deficits. Over the years, clinical and genetic studies have demonstrated that CMT is extremely heterogeneous. A classification was proposed in the 1970s aiming to group the most common CMT variants as hereditary motor and sensory neuropathies (HMSN). In CMT type 1 the myelinating Schwann cells are affected, while axons are degenerated in CMT2. Besides these two autosomal dominant inherited CMT types, recessive and X-linked demyelinating and axonal CMT subtypes have been described and also included in the HMSN classification [3]. Depending on the severity of motor or sensory deficiency, other CMT variants were grouped into predominantly distal hereditary *motor* neuropathies (distal HMN) and hereditary *sensory and autonomic* neuropathies (HSAN) [4,5]. More recently, clinical and genetic overlaps have been reported between CMT neuropathies and hereditary spastic paraplegias. In addition, there have been cases with more complex clinical phenotypes involving other tissues, such as skin and bone (reviews by [6,7,8]), further complicating the original CMT classification. 

**Figure 1:**
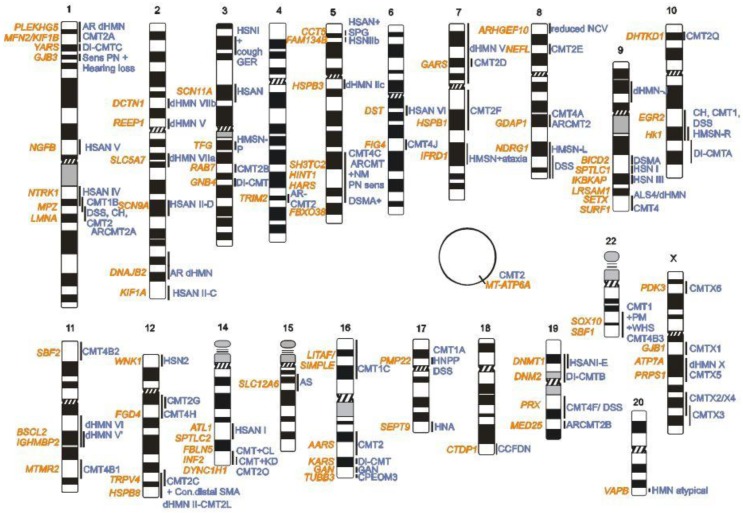
Genes and loci for Charcot-Marie-Tooth (CMT) and related inherited peripheral neuropathies.

**Figure 2:**
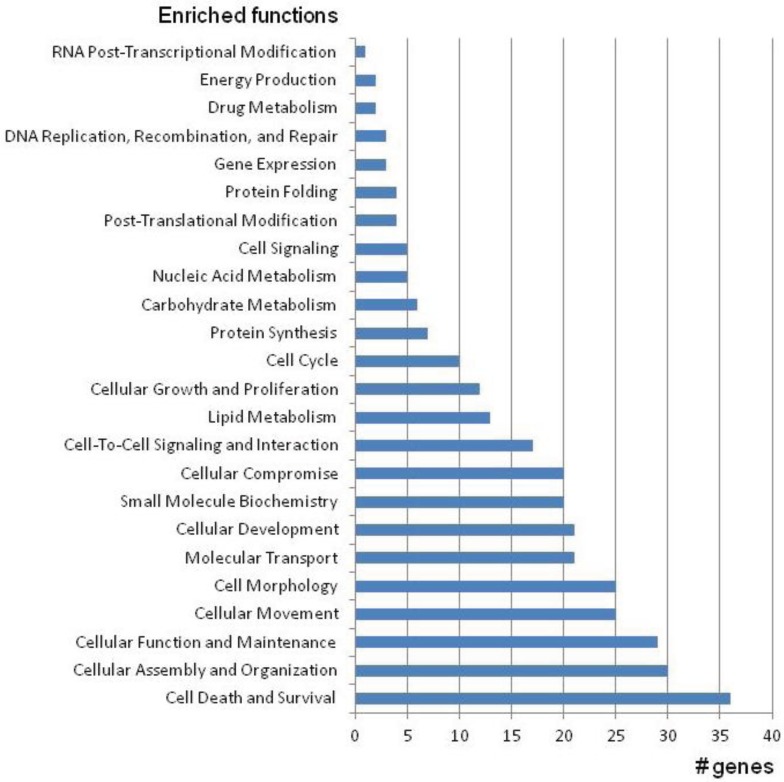
Functional categories containing enriched molecular and cellular functions of genes involved in CMT and related neuropathies.

The first CMT locus was mapped in 1982 [9], and 30 years of genetic research has not only allowed the successful identification of 80 disease-causing genes, but also pioneered the discovery of novel genomic mechanisms (Figure 1). Loci and genes for CMT and related peripheral neuropathies were initially identified using genetic linkage studies, positional cloning, or candidate gene approaches. Since the publication of the Human Genome in 2001 [10,11], the development of high-throughput technologies, such as whole genome mapping (WGM), whole genome sequencing (WGS), and whole exome sequencing (WES) [12,13] accelerated the gene and mutation discovery in CMT research. Genetic research in CMT has shown that all Mendelian inheritance patterns are possible. However, besides dominant, recessive, and X-linked inherited CMT types, mutations also occur *de novo* in isolated patients. More recently, a CMT phenotype was associated with a defect in *MT-ATP6A*, a gene encoded by the mitochondrial DNA [14]. Different CMT phenotypes can be caused by mutations in the same gene, and conversely mutations in different genes may result in the same phenotype. This is further complicated by the fact that some mutations are extremely rare and occur in specific subtypes. Mutations in more than 20 genes cause primary alterations of the myelin sheath; well-known examples include *MPZ*, *PMP22*, and *GJB1*. Mutations in genes with axonal functions, however, result in axonal CMT and associated phenotypes (e.g., *NEFL*, *GAN*). Their gene products have cell-type specific functions, allowing underlying disease mechanisms to be logically inferred. Other mutations have been reported to cause intermediate CMT, with both myelin and axonal phenotypes. The availability of the Human Genome also contributed to the identification of mutations in genes that were not the primary functional candidates for CMT neuropathies. Examples include mutations found in ubiquitously expressed genes coding for amino-acyl tRNA synthetases (*GARS*, *YARS*, *HARS*, *MARS*, *AARS*), small heat shock proteins (*HSPB1*, *HSPB3*, *HSPB8*) and enzymes involved in membrane and transport metabolism (*SPTLC1*, *SPTLC2*, *MTMR2*, *SBF1*, *SBF2*), whose resulting gene products have housekeeping functions and pleiotropic activities. In addition, CMT disease-associated genes are expressed in different cellular compartments of the developing and myelinating Schwann cells and/or the neuronal axons [15]. Some of these genes have been shown to function in the nucleus as transcription factors (*EGR2*, *SOX10*, *DNMT1*), others in vesicle transport (*RAB7A*), in the Golgi (*FAM134B*), endoplasmic reticulum (*SPTLC1*, *REEP1*, *ATL1*), or the mitochondria (*MFN2*, *GDAP1*). For most of these genes, it still remains an enigma why the mutant proteins cause such specific, length-dependent degeneration of peripheral nerves in CMT patients (Figure 2).

The figure shows 80 currently known genes (orange symbols) and their corresponding chromosomal loci (vertical bars). The corresponding phenotypes are indicated by blue symbols and are according to the disease nomenclature. Note that the disease names may not always correspond to information available in OMIM, GeneReviews, or in other publicly available databases. The full names of the gene symbols and year of gene identification are provided in Supplementary Table S1.

Ingenuity Pathway Analysis (IPA version 17199142, Ingenuity Systems [16]) was used to summarize the molecular and cellular functions that were most strongly associated with genes linked to inherited peripheral neuropathies. Detailed gene functions are provided as Supplementary Tables S2. Corresponding *p*-values in the supplementary files were calculated using Fisher’s exact test and corrected for multiple testing using the Benjamini-Hochberg method. Note that the same gene can be present in various clusters.

**Figure 3:**
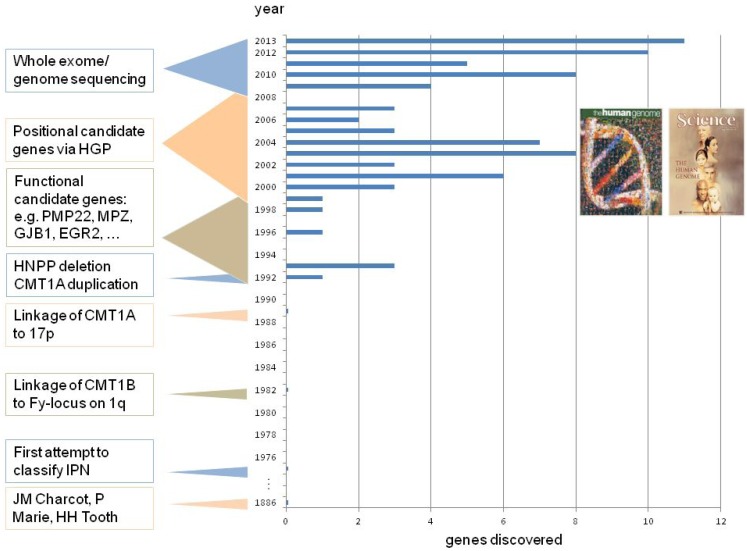
Historical overview of gene identification in CMT and related inherited peripheral neuropathies.

The tremendous success of molecular genetics between 1990 and 2004 can be attributed to multiplex studies of large CMT families suitable for positional cloning and candidate gene screening. Because such families have become rare, the pace of gene discovery slowed down soon after the publication of the Human Genome (Figure 3). Reflecting the clinical reality, the majority of patients with peripheral neuropathies derive from nuclear families or represent isolated patients with severe phenotypes. Despite their huge potential, these patients and nuclear families were beyond the reach of classical gene discovery approaches. Fortunately, this situation has changed spectacularly with the introduction of novel, affordable sequencing technologies which allow massive, genome-wide analysis of entire exomes (all protein coding regions) or even genomes. We will discuss the history of CMT gene discoveries by providing a few highlights where the Human Genome Project (HGP) contributed to the gene finding. As not all discoveries can be discussed, we provide a comprehensive table listing all currently known disease-causing genes for CMT, as well as the original technologies used to find the associated genes and mutations (Supplementary Table S1). Further details can be obtained from corresponding references to the literature, via the OMIM database [17], IPNMDB database [18], or LOVD database [19], which in part provide a list of mutations and genetic variants. 

This figure shows the number of genes found per year since the identification of the CMT1A duplication in 1991. Note a peak in gene discovery soon after the publication of the HGP in 2001. A second peak occurs when next generation sequencing (NGS) tools became available from 2009. We also highlighted the introduction of NGS methods (WES and WGS), as well as the major functional and positional CMT genes identified before and after the publication of the HGP. All genes identified per year are listed in Supplementary Table S1.

## 2. CMT Genetics as a Pioneer for Genomic Mechanisms and Emerging Genome Technologies

### 2.1. Early Linkage Studies

In 1982, T. Bird assigned the first locus for autosomal dominant CMT by screening the Duffy-blood group marker in a family with demyelinating HMSN. He obtained genetic linkage between CMT and the Duffy locus on chromosome 1 [9], which was soon confirmed in other CMT families and defined as the CMT1B subtype (reviewed in [20]). However, the Duffy-blood group marker did not segregate in several other large CMT families which were grouped within the CMT1A subtype of HMSN [21,22,23]. It took as long as 10 years to find that the *MPZ* gene coding for the major peripheral myelin protein (P0) was mutated in CMT1B patients [24]. The *MPZ* gene was assigned to chromosome 1q22-q23 and was the perfect candidate gene for a demyelinating peripheral neuropathy. Further genetic research, based on sequencing the coding region of the *MPZ* gene, demonstrated that mainly heterozygous, missense mutations occur in CMT1B. Other rare mutations in *MPZ* were associated with severe and early onset peripheral neuropathies, such as Roussy-Levy syndrome [25], Dejerine-Sottas syndrome [26] and congenital hypomyelination [27]. At least 117 mutations have been described in *MPZ* [18], and genotype/phenotype correlations associated a few *MPZ* mutations with axonal HMSN or CMT2 [28,29]. Although CMT patients can be routinely screened for *MPZ* mutations in DNA diagnostic labs, the CMT1B subtype is less frequent than CMT1A [30,31]. 

### 2.2. CMT1A—The First ‘Genomic Disorder’

The CMT1A subtype is the most common HMSN and one of the first genetic examples of a submicroscopic genomic disorder. The CMT1A locus was assigned, in 1989, to chromosome 17 through genetic linkage studies in large HMSN families using restriction fragment length polymorphic (RFLP) markers [21,22,23,32]. These families were excluded for linkage to the CMT1B locus on chromosome 1q22-q23. A multipoint linkage study allowed refinement of the CMT1A locus to a 30 cM region on 17p11.2-p12 [33]. In 1991 a tandem-duplication of 1.4 megabases (Mb) on chromosome band 17p12 was identified as a frequent cause for CMT1A and represented 70% of CMT1 in many populations [30,31,34,35]. Several molecular methods revealed the CMT1A duplication: the presence of three informative alleles by RFLP analysis and polymorphic dinucleotide (CA)_n_ repeats in affected individuals, the identification of a patient-specific junction fragment by pulsed-field gel electrophoresis (PFGE), and the duplication of probes detected by fluorescence *in situ* hybridization (FISH) (reviewed in [36]). A deletion of the same chromosomal region in 17p12 resulted in a distinct form of inherited peripheral neuropathy, known as hereditary neuropathy with liability to pressure palsy (HNPP) [37]. Through the availability of large insert clones, such as yeast artificial chromosomes (YAC), P1 artificial chromosomes (PAC) and bacterial artificial chromosomes (BAC), it became possible to build clone contigs of chromosome 17. These clones could be further subcloned into smaller fragments, which could then undergo DNA sequencing analysis. This procedure allowed sequencing of 1,421,129 bp of DNA at the CMT1A duplication and HNPP deletion region. Furthermore, this 1.4 Mb chromosomal region was found to be flanked by two 24 kb homologous low copy repeats (LCRs) called the proximal and distal CMT1A-REPs. This unique genomic architecture creates a non-allelic homologous recombination (NAHR) which cause the CMT1A duplication or HNPP deletion. Further analysis of the genomic region nearby the CMT1A-REPs demonstrated an evolutionary mechanism for the formation of the CMT1A-REP and the creation of novel genes by DNA rearrangement [38,39,40]. The *PMP22* gene, encoding the peripheral myelin protein 22, was physically assigned in the middle of the 1.4-Mb CMT1A region using somatic chromosomal hybrid cell lines, PFGE restriction, and YAC maps [41,42,43,44]. As a consequence, one additional copy of *PMP22* is responsible for CMT1A, whereas loss of one copy of *PMP22* results in HNPP, highlighting a gene dosage effect as the mechanism for these disorders [45]. In addition, some CMT1A and HNPP neuropathy patients have apparent rare copy number variations (CNVs) of an atypical size in the 17p12 region [46]. More recent, detailed analysis of these genomic rearrangements by high-density, oligonucleotide-based array comparative genomic hybridization (aCGH) and subsequent sequencing of the CMT1A/HNPP breakpoint revealed non-recurrent rearrangements including: non-homologous end joining (NHEJ), *Alu-Alu*-mediated recombination, and DNA replication-based mechanisms such as fork stalling and template switching (FoSTeS) and microhomology-mediated break-induced replication (MMBIR) [47]. All these studies confirmed that *PMP22*, either altered by dosage or dysregulation, is the major gene responsible for CMT1A and HNPP. The identification of the CMT1A duplication and reciprocal HNPP deletion on 17p12 has also shown that rare CNVs involving both coding and non-coding sequences can cause human disorders (reviewed in [48,49]). Further genetic research in CMT and HNPP resulted in the identification of 61 different point mutations in *PMP22*.

Some of these *PMP22* mutations have been described in naturally occurring mouse mutants (trembler mouse) or have been modeled in transgenic animals [50,51] (reviewed in [52]). Rodent models expressing multiple copies of the *PMP22* gene mimic the human CMT1A duplication and have been instrumental in understanding the disease mechanism and developing therapeutic approaches. Anti-progesterone or ascorbic acid (Vitamin C) has been used to alleviate the typical demyelinating neuropathy in CMT1A rat and mouse models respectively [53,54]. Based on this data, multicenter clinical trials with the aim to treat CMT1A duplication patients have been developed for adults and children, but did not reveal significant improvement of the disease symptoms [55,56,57,58,59,60]. Recently, clinicians and researchers have focused on the natural history of peripheral neuropathies and developed reliable clinical and DNA diagnostic guidelines [61,62]. These internationally accepted guidelines will be important to support other treatment strategies for CMT1A currently under investigation.

### 2.3. Genetic and Physical Mapping, and the Contribution of the Human Genome Reference to Gene Finding in CMT

The HGP has significantly contributed to the identification of genes that were not considered likely candidates for peripheral neuropathies. Here we provide a research example for CMT-related neuropathies where the motor neurons are predominantly affected. The clinical characteristics of this entity, also known as distal hereditary motor neuropathy (HMN), have been defined by A. Harding and P.K. Thomas in 1980 [63]. The identification of the distal HMN genes also started through genetic linkage studies in extended families in which the disease was dominantly transmitted (reviewed in [64]). These studies were labor intensive and time consuming due to the limited availability of genetic markers, which were mainly RFLPs analyzed by Southern blotting and hybridization with radioactive labeled probes. Linkage excluded the CMT1A and CMT1B loci on chromosomes 17 and 1 respectively [65]. However, thanks to the discovery of highly polymorphic short tandem repeat (STR) markers and their detection through PCR methods, genome-wide scans (GWS) allowed the identification of one of the first distal HMN loci on chromosome 12. The GWS was performed with a multiplex procedure for genotyping microsatellite markers (referred to as *afm-markers*) combined with a hybridization-based detection technology [66]. A total of 187 (CA)_n_ repeat polymorphisms located on chromosomes 1 to 12 were genotyped at a mean distance of 15 cM. Based on the segregation analysis of STR alleles, the presence of informative recombinants, and multipoint linkage analysis, a candidate region for the distal HMN gene was delineated to a region of 13 cM [67]. Although a large part of chromosome 12 was then assembled into integrated physical, genetic, and cytogenetic maps, the distal part of 12q, including the critical region of the distal HMN locus, was not yet converted into a high-density contig. Attempts to construct a contiguous YAC-based map of the chromosomal region were not successful due to the presence of gaps or chimeric YAC clones. However, the use of PAC and BAC clones, which contained few deletions and were rarely chimeric, allowed the generation of a complete PAC/BAC contig. The PAC and BAC libraries were screened with known STR markers as well as with markers derived from cloned end-fragments of PACs and BACs using STS content mapping, PFGE analysis, Southern blotting, and fiber fluorescence *in situ* hybridizations (FISH). This final clone contig of 12q24 allowed mapping candidate genes or expressed sequenced tags (ESTs) within the critical distal HMN region [68]. The combination of genetic linkage studies (including haplotype analysis of polymorphic markers and the identification of recombinants) and the availability of the clone contig allowed further reduction of the locus from 5 to 1.7 Mb. From this refined region, known genes were selected from the HGP data [10,11] for Sanger sequencing using one of the first ABI automated DNA sequencing machines. In two large distal HMN families, a missense mutation (K141N) was found in the *HSPB8* gene coding for the 22-kDa small heat shock protein B8 (HSP22/HSPB8). In two other distal HMN families, another mutation targeted the same lysine residue (K141E) in the HSPB8 protein [69]. Interestingly, a very similar strategy resulted in the identification of another CMT locus on chromosome 7q11-q21 (CMT2F) in a large family with autosomal dominant axonal CMT [70]. A missense mutation (S135F) in the *HSPB1* gene encoding the 27-kDa small heat-shock protein B1 (HSP27/HSPB1) segregated in this CMT2F family. Screening for *HSPB1* mutations in additional CMT and HMN families confirmed the previously observed mutation and identified several additional missense mutations [71]. Both small heat shock proteins act as molecular chaperones but are also involved in many essential cellular processes such as apoptosis, autophagy, splicing, cytoskeleton dynamics, and neuronal survival (reviewed by [72]). Transgenic mouse models for mutant *HSPB1* have been created, which develop neurological symptoms similar to the human condition. Alpha-tubulin is less acetylated in sciatic nerves of mutant *HSPB1* mice when compared to wild type animals, and treatment with HDAC inhibitors (which avoids deacetylation of tubulin), ameliorated the axonal degeneration in the *HSPB1* mutant mouse [73]. Studies aimed at developing better treatment strategies for this group of axonal CMT are being tested in cell and animal models.

### 2.4. CMT2A—The Importance of a Finished Human Genome Reference

The chromosomal locus for the first axonal form of CMT was mapped to chromosome 1p36 in 1993 [74]. Despite a sizable number of mapped, large families and efforts to identify the underlying gene at multiple laboratories, no gene was discovered for over 10 years. What complicated the search was an incomplete map for chromosome 1. Within the established linkage region existed a gap of unknown size and content. In 2001, an elegant study involving cell and mouse models of *Kif1b* showed mitochondrial transport deficiencies due to loss-of-function mutations [75]. A single small CMT2 family from Japan with suggestive linkage to 1p35-36 [74] was reported to carry a specific Q98L missense mutation that showed functional deficits in a cell culture-based assay. The fact that a mutation in the motor protein *Kif1b* can underlie a peripheral neuropathy led to the conclusion that *KIF1B* is the long sought after CMT2A gene [75]. Subsequent mutation screening studies of linked CMT2A families, however, came back empty-handed. To our knowledge, no additional *KIF1B* mutations have been reported in the literature. This raised the possibility of a second gene in the region. With steadily improving genomic maps of chromosome 1, an international collaboration eventually identified mutations in the gene coding for *MFN2* in all previously linked CMT2A families [76]. *MFN2* is now established as the most common CMT2 gene accounting for ~20% of all axonal cases. Amongst the most severe and early onset forms of CMT2, *MFN2* carries a mutation in ~90% of cases [77]. The *MFN2* screening has also revealed a broader phenotypic spectrum that includes early- and late-onset cases of HMSN [78], severe and mild manifestation of symptoms [79,80] accompanying optic atrophy (HMSN VI) [81], and involvement of upper motor neurons (HMSN V) [82]. Rarely, a recessive/co-dominant trait is possible in *MFN2* [77,83].

## 3. Next Generation Sequencing Boosted the Identification of CMT Associated Genes

### 3.1. Targeted Next-Generation Sequencing and Its Limitation in CMT Gene Finding

As next generation sequencing (NGS) platforms become more and more accessible and affordable, many CMT laboratories are shifting their research towards smaller families and isolated patients who still represent a large group of genetically unsolved patients. These revolutionary technologies will allow studying isolated patients with severe phenotypes that, until recently, were beyond our reach.

Multiple studies have successfully combined whole-genome SNP genotyping, subsequent target capturing, and parallel sequencing. This approach revealed a single novel missense variant in *FBLN5* causing autosomal dominant CMT with hyperelastic skin and age-related macular degeneration [84]. A similar approach, combining whole-genome SNP genotyping, homozygosity mapping and NGS, allowed the identification of mutations in *HINT1* causing an autosomal recessive axonal neuropathy with neuromyotonia [85]. Other examples of NGS of CMT genes, with or without additional clinical features, are provided in Supplementary Table S1. As whole exome and whole genome studies become more affordable, such targeted studies will not be competitive in the near future. Furthermore, the current limitation of targeted NGS is the lack of complete coverage of some genes and the inability to detect non-exonic mutations and copy number variations (CNVs) [86,87]. 

Importantly, current diagnostic sequencing of disease genes heavily relies on targeted NGS-based methods. By creating gene panels that includes all known CMT genes, the cost of sequence production can be radically reduced, and for the first time, clinicians will have a comprehensive view of the mutational load in all CMT genes. This technique allows for a much better characterization of genotype/phenotype correlations. It will likely also uncover digenic and other unusual mutational mechanisms. The phenotypic spectra of each CMT gene will be comprehensively defined over the coming decade. At the moment a technical drawback for this approach is the less-than-100% coverage of a sequence of interest. It is expected, however, that the technology will soon match and outperform traditional Sanger sequencing in sensitivity and specificity. 

### 3.2. Whole Exome Sequencing as a Successful Approach in CMT Gene Finding

Whole exome sequencing (WES), aiming to sequence an abbreviated version of the entire genome, has become a powerful and cost-efficient method. CMT research was one of the earliest adaptors of this new technology. Montenegro *et al.* reported a study of a large CMT pedigree and the identification of a known *GJB1* mutation for the X-linked variant of CMT [88]. This was somewhat unexpected, as the family pedigree initially appeared to exclude an X-linked trait. After mutation identification, it appeared that a distant branch of the pedigree with male-to-male transmission was never clinically evaluated and likely carried a different phenotype. This study further detailed the challenges of data interpretation and incompleteness of sequence coverage of coding sequences, as well as possible strategies to resolve these shortcomings of WES. As discussed below, this approach is now well established in many clinical laboratories, only two years later.

One can now analyse whole exomes in trios (patients and their parents) for *de novo* dominant mutations. Examples of novel genes for CMT and related inherited peripheral neuropathies discovered solely based on WES include MARS [89], BICD2 [90,91,92], PDK3 [93], SCN11A [94], SLC5A7 [95], and TUBB3 [96] (Supplementary Table S1). The gene discovery rate will increase to as much as one new gene per month in the CMT field alone, until the majority of rare genes have been identified. However, WES may still be hindered by the lack of complete coverage of some genes [86,87]. Regardless, it is widely expected that these new genes will allow for a precise delineation of pathways that are key to the pathogenesis of CMT and related axonopathies. 

### 3.3. First Whole-Genome Sequencing of a CMT Patient

The first whole-genome sequence (WGS) of a CMT patient was published in 2010 [97]. This study demonstrated for the first time in all of medicine that WGS can identify clinically relevant variants and provide diagnostic information [98]. The DNA sample of the index patient belonged to a family with recessive CMT and was sequenced on the SOLiD (Sequencing by Oligonucleotide Ligation and Detection) next-generation-sequencing platform developed by Applied Biosystems. The accuracy in sequencing of 50-base reads on the SOLiD system exceeded 99% and 12 multiple sequences were read simultaneously. Overlapping reads increased the overall sequence accuracy and reduced the risk of obtaining false positive sequence variants. In the patient sample a compound heterozygous mutation was identified in *SH3TC2*, a previously known gene for recessive CMT [97,99]. The two mutations in *SH3TC2* co-segregated with the CMT disease phenotype in the pedigree. All four affected individuals had slowed nerve conduction velocities, which is indicative of a demyelinating CMT phenotype. Interestingly, the Y169H mutation also seemed to co-segregate with an electrophysiologically defined axonal neuropathy phenotype that was evident in the four affected siblings as well as the proband’s father and grandmother. By contrast, the first R954X variant in *SH3TC2* was associated with subclinical electrophysiological evidence of carpal tunnel syndrome, regardless of the presence of the second R169H mutation. Although some of the proband’s family were shown to have one or the other of these mutations, only the proband and his three siblings, who were also diagnosed with CMT, had both mutations [97]. These observations underline the importance of careful phenotyping for the valid interpretation of genomic variant data. In their study, J.R. Lupski and colleagues [97] identified over 9,000 non­synonymous single nucleotide variants (SNVs), 148 of which involved stop codons, and 112 of which were located in conserved exon splice-sites, which presumably had severe consequences for the affected proteins. Moreover, 21 of these changes were previously described as causing a Mendelian disease other than CMT. Thus, the identification of phenotypically relevant variations by means of WGS can be difficult. At the time, the authors estimated the cost of their study at ~$50,000—the same study today, three years later, would amount to less than $10,000.

## 4. Future Perspectives and the Need to Share Large Datasets and Genetic Variants

In the early days of molecular genetics, access to rare, large families was a prerequisite for linkage studies and positional cloning strategies. With the introduction of WGS and WES, this hurdle has largely been cleared. Today these novel high-throughput technologies allow the simultaneous analysis of approximately 20,000 genes in the human genome in an unbiased way. Besides the tremendous generation of DNA sequence data from complete genomes or exomes, these emerging NGS technologies also permit geneticists to tackle phenotypes that were previously largely inaccessible via Sanger sequencing. Because these methods are so powerful, NGS projects are shifting towards nuclear families and isolated patients, representing a very large group of genetically undefined patients. For each nuclear family, one can sequence two affected individuals and search for variants in genes that are shared between patients. Different strategies can be applied to study isolated patients; when the individuals are severely affected, this can be the consequence of a *de novo* mutation, and by sequencing both parents and the patient, *de novo* variations can be identified. Another approach involves sequencing unrelated index patients and detecting variations in the same gene in different individuals across families, which is a very strong and independent argument in favor of a pathogenic link between a certain gene and the CMT neuropathy. To this end, novel, innovative genome data analysis platforms have emerged, such as Genomes Management Application (GEM.app) [100]. GEM.app allows laboratories around the world to analyze their data jointly, collaborate *ad hoc* on specific novel genes, or establish networks of collaboration. This is possible via a strictly web-based system with secure access to data [101]. Every user has full control over their own data, but also sees counts of variants by gene, phenotype and variant type for all exomes/genomes in this system. The majority of novel CMT and HSP (hereditary spastic paraplegia) genes are currently discovered via GEM.app by over 200 users from 24 different countries. A variation of this approach is the Genome Variant Database for Human Diseases [101], which is heavily biased towards axonopathies. This system contains more than 500 exomes that can be freely queried to search for a “second family” to support a new gene.

Large scale screening of patients allows determination of CMT mutation frequencies, establishment of phenotypic borders of these heterogeneous neuropathies, and at the same time, exploration of phenotypic overlaps between CMT and other neuropathies. As such, NGS will be an important tool for personalized and preventive medicine. Several database initiatives aim at capturing more complete lists of clinically relevant mutations in human diseases. These include the Leiden Open Variant Database [19], the Human Gene Mutation Database [102], and a specific CMT database currently constructed by the Inherited Neuropathy Consortium [103] However, CMT genetics have already identified more than 1,000 mutations in 80 disease associated genes, and novel NGS tools will unravel at least an equal amount of CMT associated genes, making it more appropriate to study the common disease mechanisms. Importantly, the development of NGS technologies also led to the discovery of novel mutations in known genes, uncovering their phenotypic spectrum and highlighting pleiotropic effects. 

Finally, we cannot forget the important role of functional studies in unraveling gene and protein functions, and in particular the study of mutations in cell and animal models [104]. In general, these studies were designed to understand the complex pathomechanisms of axonal degeneration and myelination defects in the peripheral nervous system. Cell and animal models that have been developed for a large group of peripheral neuropathy associated genes will be instrumental for treatment of CMT and related disorders [105]. However, focusing on treatment strategies for axonal degeneration or demyelination, or aiming at treating motor and sensory defects, might be more relevant than aiming at rescuing all CMT mutations individually.

## Supplementary Files

Supplementary File 1 Supplementary Table 1 (PDF, 339 KB)

Supplementary File 2 Supplementary Table 2 (XLS, 65 KB)
