# Supplementary material for: Genetics of Charcot-Marie-Tooth (CMT) Disease within the Frame of the Human Genome Project Success
Source: Genes (Basel). 2014 Jan 22;5(1):13–32. doi: 10.3390/genes5010013 (PMC3978509; doi:10.3390/genes5010013)
Supplement: Supplementary File 1 — Supplementary Table 1 (PDF, 339 KB) [file genes-05-00013-s001.pdf]

Supplementary Information

Table S1. Original technologies used to find the associated genes and mutations for CMT and related inherited peripheral neuropathies.

Chromosomal region	Gene symbol	Gene name	Peripheral neuropathy phenotype(s)	Type of mutation(s)	Original technologies used to find the disease associated gene/mutation(s)	Key refs	OMIM Entry	Year of gene identification	Mutations known so far
1p36.31	PLEKHG5	Pleckstrin homology domain-containing, family G member 5	RI-CMT	compound heterozygous and homozygous mutations	WES, homozygosity mapping with ABI-Prism LMS 2, Sanger confirmation of PLEKHG5 based on connection with CMT and lower motor neuron disease databases	[1,2]	611101	2013	3
1p36.2	KIF1B	Kinesin family member 1B	CMT2A1	missense mutation (Q98L)	sequencing of KIF1B based on a mouse model	[3]	605995	2001	1
1p36.2	MFN2	Mitofusin 2	CMT2A, HMSN-V	dominant mutations	STR markers, linkage analysis, sequencing of positional candidate genes, exclusion of KIF1B	[4,5]	608507	2004	50
1p34-p35	YARS	Tyrosyl-tRNA synthetase	DI-CMTC	dominant mutations	STR markers, linkage analysis, exclusion of candidate genes by sequencing	[6,7]	603623	2006	3
1p34	GJB3 (Cx31)	Gap junction protein B3, Connexin 31	sensory neuropathy + hearing loss	point mutation (D66Del)	candidate gene analysis by sequencing	[8]	603324	2001	1
1p11.2-p13.2	NGFB	Nerve growth factor beta	HSAN-V	dominant mutations	genome-wide screen and homozygosity mapping using ABI 10 cM SNP mapping panel, exclusion of positional candidate genes by sequencing	[9]	162030	2004	2
1q21-q22	NTRK1 (TRKA)	Neurotrophic tyrosin kinase receptor 1	HSAN-IV, CIPA	recessive mutations	candidate gene analysis by sequencing	[10]	191315	1996	50
1q22-q23	MPZ	Myelin protein zero	CMT1B, CMT2, DSS, CH	dominant mutations	Duffy-blood group marker, linkage analysis, Sanger sequencing	[11,12]	159440	1993	117
1q21.2-q21.3	LMNA	Laminin A/C	AR-CMT, CMT2B1	recessive mutations	homozygosity mapping with STR markers, sequencing analysis of candidate genes	[13]	150330	2002	3
2p13.1	DCTN1	Dynactin 1	dHMN-VIIb	dominant mutation (G59S)	genome-wide screen using ABI-Prism LMS 2 and sequencing of positional candidate genes	[14]	601143	2003	1

Table S1. *Cont.*

**Chromosomal region**	**Gene symbol**	**Gene name**	**Peripheral neuropathy phenotype(s)**	**Type of mutation(s)**	**Original technologies used to find the disease associated gene/mutation(s)**	**Key refs**	**OMIM Entry**	**Year of gene identification**	**Mutations known so far**
2p11.2	REEP1	Receptor expression enhancing protein 1	dHMN-Vb	dominant splice-site mutation (c.304-2A > G)	multipoint linkage analysis by applying Affymetrix GeneChip Human Mapping 10 K arrays and WES by Genome Analyzer HiSeq 2000 system	[15]	609139	2012	1
2q14	SLC5A7	Solute Carrier Family 5 (Choline Transporter), Member 7	dHMN-VIIa	dominant mutation c.1497delG (Lys499Asnfs ×13)	linkage analysis with ABI-Prism LMS 2 and WES by capturing with SureSelect All Exons (50 Mb) and sequenced by Illumina HiSeq	[16,17]	608761	2012	1
2q24.3	SCN9A	Sodium channel protein type 9 subunit alpha	HSAN II-D	recessive mutations	screen for known HSAN causative and related genes, confirmation by Sanger sequencing	[18,19]	603415	2013	2
2q34-q36.1	DNAJB2 (HSJ1)	DnaJ (Hsp40) homolog, subfamily B, member 2	AR-dHMN	homozygous splice-site mutation (c.35211G > A)	homozygosity mapping strategy (DeCode Genetics) and sequencing of candidate genes	[20]	604139	2012	1
2q37.3	KIF1A	Kinesin family member 1A	HSAN-II-C	recessive mutations	yeast-two-hybrid screen combined with genome-wide homozygosity mapping using the Illumina HumanHap300-Duov2 Genotyping BeadChip and DNA sequencing using the 3730XL DNA analyzer	[21]	601255	2011	2
3p22.2	SCN11A (NAV1.9, NaN)	Sodium channel, voltage-gated, type XI, alpha	HSAN with loss of pain perception	*de novo* missense mutations	WES of trios on an Illumina platform, validation of the variants by Sanger sequencing	[22]	604385	2013	2
3p22-p24	unknown	unknown	HSN-I with cough and gastroesophageal reflux	dominant inheritance	genome-wide scan and linkage analysis	[23,24]			

Table S1. *Cont.*

Chromosomal region	Gene symbol	Gene name	Peripheral neuropathy phenotype(s)	Type of mutation(s)	Original technologies used to find the disease associated gene/mutation(s)	Key refs	OMIM Entry	Year of gene identification	Mutations known so far
3q12	TFG	TRK-Fused Gene Protein	HMSN-I, proximal	dominant missense mutation (P285L)	Genome-Wide Human SNP array 6.0 (Affymetrix) followed by Sequence Capture Human Exome 2.1 M Array (NimbleGen)	[25]	602498	2012	1
3q21.3	RAB7	Small GTPase Rab7	CMT2B	dominant missense mutations	STR markers, linkage analysis, sequencing of positional candidate genes	[26,27]	602298	2003	5
3q26.3	GNB4	Guanine nucleotide binding protein (G protein), beta polypeptide 4.	DI-CMTF	dominant missense mutations	genome-wide linkage analysis and subsequent exome sequencing, validation of the variants by Sanger sequencing	[28]	610863	2013	2
4q31.3	TRIM2	Tripartite motif containing 2	AR-CMT2	compound heterozygous mutations	WES using NimbleGen Sequence, Capture 2.1M Human Exome v2.0 array and sequencing with Illumina Genome Analyzer-IIx platform	[29]	614141	2013	2
5p15.31-p14.1	CCT5	Chaperonin containing TCP1, subunit 5	HSAN with spastic paraplegia	homozygous missense mutation (A492G)	homozygosity mapping with STRs, sequencing analysis	[30,31]	610150	2006	1
5p15.1	FAM134B	Family with sequence similarity 134, member B	HSN-IIb	recessive mutations	genome-wide homozygosity mapping using Affymetrix GeneChip Human Mapping 50K and subsequently sequencing analysis of candidate genes	[32]	613114	2009	4
5q11.2	HSPB3	Small heat shock protein B3	dHMN-IIc	dominant missense mutation (R7S)	candidate gene approach based on identification of mutations in small heat shock proteins and sequencing	[33]	604624	2010	1
5q23-q33	SH3TC2	SH3 domain and tetratricopeptide repeats-containing protein 2	CMT4C	recessive mutations	homozygosity mapping strategy and sequencing of positional candidate genes	[34,35]	608206	2003	19

Table S1. *Cont.*

Chromosomal region	Gene symbol	Gene name	Peripheral neuropathy phenotype(s)	Type of mutation(s)	Original technologies used to find the disease associated gene/mutation(s)	Key refs	OMIM Entry	Year of gene identification	Mutations known so far
5q31.1	HINT1	Histidine triad nucleotide-binding protein 1	AR-CMT with neuromyotonia	recessive mutations	homozygosity mapping strategy using the Illumina Human660W-Quad platform and Affymetrix Human Mapping 50 K Xba array, and paired-end sequencing by Complete Genomics	[36]	601314	2012	8
5q31.3	HARS	Histidine-tRNA synthase	PN with sensory symptoms	missense mutation (R137Q)	candidate gene approach using WES	[37]	142810	2013	1
5q33.1	FBXO38	F-box protein 38	Distal SMA with calf predominance	dominant mutation (Cys206Arg)	genetic linkage analysis and exome sequencing	[38]	608533	2013	1
6p12.1	DST	Dystonin	HSAN-VI	recessive mutation (A4956LfsX26)	homozygosity mapping using the Affymetrix GeneChip Human Mapping 250 K Nsp Array and exome sequencing with SureSelect Human All Exon v.2 Kit (Agilent)	[39]	113810	2012	1
6q21	FIG4	SAC domain-containing protein gene Fig4	CMT4J	recessive mutations	mapping of mutation in pale tremor mouse (microsatellite and SNP markers, sequencing of candidate genes), sequencing of Fig4 in patients lacking mutations in known genes	[40]	609390	2007	4
7p14.3	GARS	Glycyl-tRNA synthetase	CMT2D, dHMN-V	dominant mutations	STRs, linkage analysis, sequencing of 11 candidate genes mapping in the critical region	[41–45]	600287	2003	10
7q11.23	HSPB1 (HSP27)	Small heat shock protein B1	CMT2F, dHMN2B	dominant and recessive mutations	fluorescent Human Gene Mapping Kit (Weber set 6), linkage analysis with STR markers, sequencing of positional candidate genes	[46,47]	602195	2004	6

Table S1. *Cont.*

Chromosomal region	Gene symbol	Gene name	Peripheral neuropathy phenotype(s)	Type of mutation(s)	Original technologies used to find the disease associated gene/mutation(s)	Key refs	OMIM Entry	Year of gene identification	Mutations known so far
7q31.1	IFRD1	Interferon-related developmental regulator 1	HMSN with ataxia	dominant missense mutation (I172V)	linkage (ABI Prism LMS 2 and custom primer sets), evaluation for nucleotide repeat expansions (UCSC GB simple repeats), array CGH to identify microdeletions and -duplications, sequencing of candidate genes (NimbleGen capture array, Illumina Genome Analyzer I sequencer)	[48,49]	603502	2009	1
8p23.3	ARHGEF 10	Rho guanine nucleotide exchange factor (GEF) 10	PN with reduced nerve conduction	dominant missense (T109I)	genome-wide linkage, haplotype analysis, sequencing of positional candidate genes	[50]	608136	2003	1
8p21.2	NEFL	Neurofilament light chain	CMT2E	dominant mutations	microsatellite markers to test linkage with known CMT loci, genome-wide linkage, SSCP mutation screening of positional candidate genes, sequencing gene exon of interest	[51]	162280	2000	30
8q21.11	GDAP1	Ganglioside-induced differentiation-associated protein 1	CMT4A, ARCMT2, CMT4D	recessive mutations	linkage analysis with microsatellites, YAC/PAC/BAC contig mapping to refine the region, using microsatellites and STSs, SSCP mutation screening to reject PMP22 as culprit gene, gap-filling with sequence from the Human Genome Draft Sequence, sequencing of candidate genes, homozygosity mapping	[52–54]	606598	2002	29

Table S1. *Cont.*

Chromosomal region	Gene symbol	Gene name	Peripheral neuropathy phenotype(s)	Type of mutation(s)	Original technologies used to find the disease associated gene/mutation(s)	Key refs	OMIM Entry	Year of gene identification	Mutations known so far
8q24.22	NDRG1	N-myc downstream-regulated gene 1	HMSN-Lom	recessive mutations	Analysis for segment sharing (Research Genetics Genome Screening Set 4), linkage across chromosome 8q (Genethon markers), BAC/PAC contig sequencing, sequencing of targeted region	[55,56]	605262	2000	2
8q23-q24	unknown	unknown	DSS	dominant inheritance	linkage analysis	[57]			
9p21.2-p12	unknown	unknown	dHMN-Jerash	recessive inheritance	genome-wide homozygosity mapping	[58,59]			
9q22.31	BICD2	Bicaudal D homolog 2	DCSMA	dominant mutations	genome-wide linkage (Illumina SNP), exome sequencing (Agilent SureSelect v.2, Illumina HiSeq 2000), variant filtering, confirmation of variants found in other families	[60–62]	609797	2013	4
9q22.31	SPTLC1	Serine palmitoyl-transferase, long chain base subunit 1	HSN-I	dominant missense mutations	linkage analysis, radiation hybrid mapping and physical mapping, CEPH YAC clones and EST content mapping, cDNA cloning, Sanger sequencing to confirm the mutations	[63–67]	605712	2001	7
9q31.3	IKBKAP	Inhibitor of kappa light polypeptide gene enhancer in B-cells, kinase complex-associated protein	HSN-III	recessive mutations	linkage with RFLP and STR markers, cDNA cloning and cDNA library screen, SSCP mutation analysis, cosmid exon-trapping, sequencing of the gene	[68–70]	603722	2001	3

Table S1. *Cont.*

Chromosomal region	Gene symbol	Gene name	Peripheral neuropathy phenotype(s)	Type of mutation(s)	Original technologies used to find the disease associated gene/mutation(s)	Key refs	OMIM Entry	Year of gene identification	Mutations known so far
9q33.3	LRSAM1	Leucine rich repeat and sterile alpha motif containing 1	CMT2 type	dominant mutations	SNP genotyping, exclusion of known CMT loci by haplotype and linkage analysis, exclusion of candidate genes, linkage analysis (Affymetrix 250 K SNP array), custom sequence capture array of region, sequence analysis (FLX Titanium sequencer Roche), segregation analysis of mutations	[71,72]	610933	2012	2
9q34.13	SETX	Senataxin	dHMN, ALS4	dominant missense mutations	linkage (Research Genetics v.6), EST content mapping on cosmids, exclusion of candidate genes by sequencing	[73–75]	608465	2004	4
9q34.2	SURF1	Surfeit locus protein 1	CMT4	recessive splice-site and compound heterozygous	exclusion of candidate genes, direct sequencing of SURF1	[76]	185620	2013	3
10p14	DHTKD1	Dehydrogenase E1 and transketolase domain containing 1	CMT2Q	dominant nonsense mutation Tyr485 *	genome-wide linkage analysis, sequencing of candidate genes	[77]	614984	2012	1
10q21.3	EGR2 (KROX20)	Early growth response gene 2	CH, CMT1D, DSS	dominant or de novo heterozygous mutations	heteroduplex analysis of EGR2, based on mouse model, direct sequencing of the gene	[78]	129010	1998	13
10q22.1	HK1	Hexokinase 1	HMSN-Russe, CMT4G	recessive missense mutations	linkage analysis to known chromosomal regions for AR-HMSN, linkage (ABI Prism LMS 1 and 2), markers from Genethon database, exclusion of EGR2 by sequencing, Sanger sequencing of exons and ESTs in candidate region	[79–81]	142600	2009	2

Table S1. *Cont.*

Chromosomal region	Gene symbol	Gene name	Peripheral neuropathy phenotype(s)	Type of mutation(s)	Original technologies used to find the disease associated gene/mutation(s)	Key refs	OMIM Entry	Year of gene identification	Mutations known so far
10q24.1-q25.1	unknown	unknown	DI-CMTA	dominant inheritance	linkage studies to exclude known CMT loci, genome-side scan (ABI Prism LMS MD-10, ABI 3700 automated sequencer), two-point linkage with STR markers	[82,83]			
11p15.4	SBF2 (MTMR13)	SET binding factor 2	CMT4B2 with early onset glaucoma	recessive mutations	haplotype reconstruction, exclusion of known loci, linkage analysis, cDNA screen, sequencing of target gene (ABI 3100 sequencer)	[84–87]	607697	2003	5
11q12.3	BSCL2	Berardinelli-Seip congenital lipodystrophy 2 (seipin)	dHMN-V, Silver Syndrome	dominant missense mutations	STRs (DNA sequencer 4000, LI-COR), exclusion of known loci, haplotype analysis with STR markers, sequencing of candidate genes in region of interest (ABI 3100 DNA Analyzer)	[88–90]	606158	2004	2
11q13.3	IGHMBP2	Immunoglobulin μ-binding protein 2	dHMN-VI (AR-SMARD, diaphragmatic SMA)	recessive mutations	genome-wide linkage (Genethon, ABI 377 Sequencer), sequencing of target gene based on mouse model	[91,92]	600502	2001	55
11q21	MTMR2	Myotubularin related protein 2	CMT4B1	recessive mutations	Southern blot hybridization to analyze duplication, SSCP mutation screening, microsatellite analysis, linkage analysis, YAC contig mapping, FISH analysis, EST sequencing (ABI 377 Sequencer), sequencing of candidate genes	[93–96]	603557	2000	18
12p13.33	WNK1 (PRKWNK1, HSN2)	WNK lysine deficient protein kinase 1	HSN2	recessive mutations	genome-wide scan with microsatellites, sequencing of candidate genes within region	[97]	605232	2004	12

Table S1. *Cont.*

Chromosomal region	Gene symbol	Gene name	Peripheral neuropathy phenotype(s)	Type of mutation(s)	Original technologies used to find the disease associated gene/mutation(s)	Key refs	OMIM Entry	Year of gene identification	Mutations known so far
12p11.21	FGD4	FYVE, RhoGEF and PH domain containing 4 (frabin)	CMT4H	recessive mutations	genome-wide screen (STR from Genethon), exclusion of candidate genes, targeted sequencing of FGD4 based on location and that it codes for a Rho GTPase	[98–100]	611104	2007	5
12q12-q13.3	unknown	unknown	CMT2G	dominant inheritance	exclusion of known genes, genome-wide linkage (ABI Prism LMS 2.5), exclusion of candidate genes	[101]			
12q24.11	TRPV4	Transient receptor potential cation channel, subfamily V	CMT2C, congenital distal SMA	dominant missense mutations	microsatellite linkage, fine mapping, haplotype reconstruction, SNT array analysis of region of interest, sequencing of all protein-coding genes within region of interest	[102–105]	605427	2010	6
12q24.23	HSPB8 (HSP22)	Small heat shock protein B8	dHMN-II, CMT2L	dominant missense mutations	genome-wide hybridization-based linkage screen (Genethon), YAC contigs, PAC/BAC contigs, EST, STS and STR content mapping, haplotype analysis with STR markers, sequencing of positional candidate genes, Sanger sequencing to confirm mutations	[106–110]	608014	2005	3

Table S1. *Cont.*

Chromosomal region	Gene symbol	Gene name	Peripheral neuropathy phenotype(s)	Type of mutation(s)	Original technologies used to find the disease associated gene/mutation(s)	Key refs	OMIM Entry	Year of gene identification	Mutations known so far
14q22.1	ATL1	Atlastin 1	HSN1	dominant missense mutations	exclusion of candidate genes, genome-wide linkage analysis (Affymetrix GeneChip Human Mapping 10 K array XbaI 142 2.0), high-throughput sequencing (NimbleGen custom tiling 385 K sequence capture array), confirmation of the variant by Sanger sequencing	[111]	606439	2010	3
14q24.3	SPTLC2	Serine palmitoyl-transferase, long chain base subunit 2	HSAN-I	dominant missense mutations	sequencing of SPTLC2 as a candidate gene based on knowledge of mutations in SPTLC1 (both genes code for subunits of the SPT enzyme)	[112]	605713	2010	4
14q32.12	FBLN5	Fibulin 5	CMT1 with cutis laxa, age-related macular degeneration	dominant missense mutations	common mutations excluded, linkage analysis (Affymetrix GeneChip Human Mapping 10 K array XbaI 142 2.0), array-based sequence capture for Chromosome 14 (custom tiling 385 K Roche NimbleGen), confirmation of the variant by Sanger sequencing	[113]	604580	2011	3
14q32.33	INF2	Inverted formin, FH2 and WH2 domain containing	CMT with focal segmental glomerulosis	dominant mutations	sequencing of INF2 based on known mutations causing focal segmental glomerulosclerosis (kidney disease) and its role in myelination	[114]	610982	2011	9
14q32.31	DYNC1H1	Dynein, cytoplasmic 1, heavy chain 1	CMT2O, SMA, mental retardation	dominant mutation (H306R)	common mutations excluded, WES (Agilent SureSelect whole-exome kit 1), confirmation of the variant by Sanger sequencing	[115]	600112	2011	1

Table S1. *Cont.*

Chromosomal region	Gene symbol	Gene name	Peripheral neuropathy phenotype(s)	Type of mutation(s)	Original technologies used to find the disease associated gene/mutation(s)	Key refs	OMIM Entry	Year of gene identification	Mutations known so far
15q14	SLC12A6 (ACCPN, KCC3)	Solute carrier family 12, member 6	HMSN, Andermann syndrome, agenesis of the corpus callosum	recessive truncations	linkage analysis (dinucleotide repeat polymorphic markers, 1993–1994 Genethon map, and DHLC database), recombination mapping, haplotype analysis, SSCP analysis	[116,117]	604878	2002	5
16p13.13	LITAF (SIMPLE)	Lipopoly-saccharide-induced TNF factor	CMT1C	dominant missense mutations	positional cloning and candidate gene approaches, Sanger sequencing	[118,119]	603795	2003	19
16q22.1	AARS	Alanine-tRNA synthetase	CMT2N	dominant missense mutation (R329H)	exclusion of candidate genes, linkage analysis using genome-wide human Affymetrix SNP array 6.0	[120]	601065	2010	1
16q23.1	KARS	Lysine-tRNA synthetase	DI-CMT	compound heterozygous mutations	sequencing-based mutation screen of amino-acyl tRNA synthetases based on mutations reported in AARS, YARS and GARS	[121]	601421	2010	3
16q24.1	GAN	Gigaxonin	GAN	recessive mutations	homozygosity mapping, BAC similarity sequence search (BLASTN), ESTs search, SSCP and Sanger sequencing	[122–124]	605379	2000	37
16q24.3	TUBB3	Tubulin, beta 3	CFEOM3	dominant mutations	linkage analysis, DHPLC mutation analysis, confirmation of the variant by Sanger sequencing	[125–127]	602661	2010	8

Table S1. *Cont.*

Chromosomal region	Gene symbol	Gene name	Peripheral neuropathy phenotype(s)	Type of mutation(s)	Original technologies used to find the disease associated gene/mutation(s)	Key refs	OMIM Entry	Year of gene identification	Mutations known so far
17p11.2	PMP22	Peripheral myelin protein 22	CMT1A, HNPP	NAHR results in a 1.4 Mb tandem duplication, 1.4 Mb deletion, rare shorter duplications or deletions comprising PMP22, dominant mutations in PMP22	segregation and linkage analysis with RFLP and STR markers, presence of 3 informative alleles or dosage of alleles, presence of junction fragments (via pulsed-field gel-electrophoresis, Southern blotting and PCR analysis), clone contig mapping, Sanger sequencing of the 17p11.2 region and of PMP22	[128–136]	601097	1991-1993	61
17q25.3	SEPT9	Septin 9	HNA	dominant mutations	linkage analysis, STR markers, use of clone contigs, confirmation of the variant by Sanger sequencing	[137–140]	604061	2005	3
18q23	CTDP1	CTD (carboxy-terminal domain, RNA polymerase II, polypeptide A) phosphatase, subunit 1	CCFDN	recessive intronic mutation (IVS6+389C− > T)	linkage analysis (ABI Prism LMS 1 and 2), recombination mapping, NQIBD, sequencing	[141,142]	604927	2003	1
19p13.2	DNMT1	DNA (cytosine-5-) -methyl-transferase 1	HSAN-I-E	dominant mutations	linkage analysis, haplotype construction, exome sequencing (Illumina GAII and Roche454), confirmation of the variant by Sanger sequencing	[143]	614116	2011	2
19p13.2	DNM2	Dynamin 2	DI-CMTB	dominant mutations	exclusion of known loci, linkage analysis (ABI Prism LMS 2), haplotype analysis, candidate gene screening based on region and domain homology	[144–147]	126375	2005	6

Table S1. *Cont.*

Chromosomal region	Gene symbol	Gene name	Peripheral neuropathy phenotype(s)	Type of mutation(s)	Original technologies used to find the disease associated gene/mutation(s)	Key refs	OMIM Entry	Year of gene identification	Mutations known so far
19q13.2	PRX	Periaxin	CMT4F, DSS	recessive nonsense mutation	homozygosity mapping (ABI Prism LMS 2), DNA pooling, targeted sequencing based on mouse homology/BAC cloning, DHPLC mutation analysis, sequence alignment of DHPLC mutants (Sequencher)	[148–150]	605725	2001	21
19q13.33	MED25 (ARC92, ACID1)	Mediator of RNA polymerase II transcription, subunit 25	AR-CMT2B2	recessive missense mutation (A335V)	SSCP screening to eliminate known genes, genome-wide screen (Genethon microsatellite markers), BAC contig map (NT_011109)	[151,152]	610197	2009	1
20q13.3	VAPB	Synaptobrevin-associated membrane protein B	HMN (atypical late-onset SMA, ALS8)	dominant mutations	STR markers and linkage analysis, sequencing of positional candidate genes	[153]	605704	2004	3
22q13.1	SOX10	SRY (sex determining region Y)-box 10	CMT1 + Pelizaeus-Merzbacher + Waardenburg-Hirschsprung	dominant mutations	direct sequencing of target gene based on evidence of Waardenburg-Hirschsprung syndrome and murine model, as well as mutation screening of other candidate genes	[154]	602229	1999	3
22q13.33	SBF1 (MTMR5)	SET binding factor 1	CMT4B3	autosomal recessive (compound heterozygote missense mutations)	exome sequencing followed by Sanger sequencing	[155]	603560	2013	2
Xp22.11	PDK3	Pyruvate dehydrogenase lipoamide kinase isozyme 3	CMTX6	dominant missense mutation (R158H)	linkage analysis (in-house X-chromosome scan), haplotype analysis, exome sequencing, confirmation of the variant by Sanger sequencing	[156]	300906	2013	1

Table S1. *Cont.*

Chromosomal region	Gene symbol	Gene name	Peripheral neuropathy phenotype(s)	Type of mutation(s)	Original technologies used to find the disease associated gene/mutation(s)	Key refs	OMIM Entry	Year of gene identification	Mutations known so far
Xq13.1	GJB1 (Cx32)	Gap junction protein B1, Connexin 32	CMTX1	dominant mutations	RFLP and VNTR markers, direct sequencing of target based on location on X-chromosome	[157–160]	304040	1993	300
Xq21.1	ATP7A	ATPase, Cu^{2+} transporting, alpha polypeptide	dHMN-X, Menkes disease	X-linked recessive mutations	linkage analysis (ABI Prism LMS), microsatellite linkage analysis, candidate gene exclusion, high resolution melting analysis, sequencing	[161,162]	300011	2010	2
Xq22.3	PRPS1	Phosphoribosyl pyrophosphate synthetase 1	CMTX5, hearing loss, optic neuropathy, Rosenberg-Chutorian syndrome	X-linked recessive mutations	X-chromosome wide linkage (48 STR markers of ABI Prism linkage mapping set version 2.5), elimination of candidate genes, sequencing of genes known to be expressed in inner ear (Morton cochlear expression database)	[163,164]	311850	2007	2
Xq22.2	unknown	unknown	CMTX2	X-linked recessive inheritance	segregation and linkage analysis of X-chromosome RFLP markers	[165]			
Xq26.1	AIF (AIFM1)	Apoptosis-inducing factor, mitochondrion-associated, 1	CMTX4, Cowchock syndrome	X-linked recessive mutation (E439V)	RFLP and microsatellite markers on X-chromosome, exome capture with SureSelect Human All Exome Kit v.1, sequencing on Genome Analyzer IIx from Illumina	[158,166,167]	300169	2012	1

Table S1. *Cont.*

Chromosomal region	Gene symbol	Gene name	Peripheral neuropathy phenotype(s)	Type of mutation(s)	Original technologies used to find the disease associated gene/mutation(s)	Key refs	OMIM Entry	Year of gene identification	Mutations known so far
Xq26-q28	unknown	unknown	CMTX3	X-linked recessive inheritance	X-chromosome RFLP markers, direct sequencing of all coding exons except ATP11C and MCF2 which were screened using an oligo-dT reverse-transcribed template, linkage with microsatellite markers, SNP genotyping using high resolution melting analysis	[165,168,169]			
mitochondrial DNA	MT-ATP6A	Mitochondiral ATPase 6A	CMT2, Leigh syndrome	heteroplasmic (L220P)	known variants were excluded (using ABI 3730xl DNA analyzer and Seqscape v.2.5 assembly) and targeted sequencing of MT-ATP6 and MT-ATP8 was performed	[170]		2012	1

The table lists 80 currently known disease causing genes for CMT and related neuropathies, as well as the original technologies used to find the associated genes and mutations. Further details can obtained from corresponding references to the literature, via the OMIM database (ncbi.nlm.nih.gov/omim). The IPNMDB database (molgen.vib-ua.be/CMTMutations/) and LOVD database (lovd.nl) provide a list of known mutations and genetic variants for most of the 80 genes.

Abbreviations to Table S1

ALS	Amyotrophic lateral sclerosis
AR	Autosomal recessive
BAC	Bacterial artificial chromosome
CCFDN	Congenital cataracts with facial dysmorphism and neuropathy
CFEOM	Congenital fibrosis of the extraocular muscles
CGH	Comparative genome hybridization
CH	Congenital hypomyelination
CIPA	Congenital insensitivity to pain and anhydrosis
CMT	Charcot-Marie-Tooth
DHPLC	Denaturing High Performance Liquid Chromatography
dHMN	Distal hereditary motor neuropathy
DI	Dominant intermediate
DSS	Dejerine-Sottas syndrome
EST	Expressed sequenced tag
GAN	Giant axonal neuropathy
HMSN	Hereditary motor and sensory neuropathy
HNA	Hereditary neuralgic amyotrophy
HNPP	Hereditary neuropathy with liability to pressure palsies
HSAN	Hereditary sensory and autonomic neuropathy
HSN	Hereditary sensory neuropathy
LMS	Linkage Mapping Set
NAHR	Non-allelic homologous recombination
OMIM	Online Mendelian Inheritance In Man database
PAC	Phage artificial chromosome
PN	Peripheral neuropathy
RFLP	Restriction fragment length polymorphism
RI	Recessive intermediate
SNP	Single nucleotide polymorphism
SMA	Spinal muscular atrophy
SMARD	Spinal muscular atrophy with respiratory distress
STR	Short tandem repeat
SSCP	Single stranded conformation polymorphism
STS	Sequenced tagged site
VNTR	Variable Number of Tandem Repeat
WES	Whole exome sequencing
YAC	Yeast artificial chromosome

References

1. Kim, H.J.; Hong, Y.B.; Park, J.M.; Choi, Y.R.; Kim, Y.J.; Yoon, B.R.; Koo, H.; Yoo, J.H.; Kim, S.B.; Park, M.; *et al.* Mutations in the PLEKHG5 gene is relevant with autosomal recessive intermediate Charcot-Marie-Tooth disease. *Orphanet J. Rare Dis.* **2013**, *8*, 104.

2. Azzedine, H.; Zavadakova, P.; Plante-Bordeneuve, V.; Vaz, P.M.; Pinto, N.; Bartesaghi, L.; Zenker, J.; Poirot, O.; Bernard-Marissal, N.; Arnaud, G.E.; *et al.* PLEKHG5 deficiency leads to an intermediate form of autosomal-recessive Charcot-Marie-Tooth disease. *Hum. Mol. Genet.* **2013**, *22*, 4224–4232.
3. Zhao, C.; Takita, J.; Tanaka, Y.; Setou, M.; Nakagawa, T.; Takeda, S.; Wei Yang, H.; Terada, S.; Nakata, T.; Takei, Y.; *et al.* Charcot-Marie-Tooth disease type 2A caused by mutation in a microtubule motor KIF1Bbeta. *Cell* **2001**, *105*, 587–597.
4. Ben Othmane, K.; Middleton, L.T.; Loprest, L.J.; Wilkinson, K.M.; Lennon, F.; Rozear, M.P.; Stajich, J.M.; Gaskell, P.C.; Rosed, A.D.; Pericak-Vance, M.A.; *et al.* Localization of a gene (CMT2A) for autosomal dominant Charcot- Marie-Tooth disease type 2 to chromosome 1p and evidence of genetic heterogeneity. *Genomics* **1993**, *17*, 370–375.
5. Züchner, S.; Mersiyanova, I.V.; Muglia, M.; Bissar-Tadmouri, N.; Rochelle, J.; Dadali, E.L.; Zappia, M.; Nelis, E.; Patitucci, A.; Senderek, J.; *et al.* Mutations in the mitochondrial GTPase mitofusin 2 cause Charcot-Marie-Tooth neuropathy type 2A. *Nat. Genet.* **2004**, *36*, 449–451.
6. Jordanova, A.; Thomas, F.P.; Guergueltcheva, V.; Tournev, I.; Gondim, F.A.; Ishpekova, B.; de Vriendt, E.; Jacobs, A.; Litvinenko, I.; Ivanova, N.; *et al.* Dominant intermediate Charcot-Marie-Tooth type C maps to chromosome 1p34-p35. *Am. J. Hum. Genet.* **2003**, *73*, 1423–1430.
7. Jordanova, A.; Irobi, J.; Thomas, F.P.; van Dijck, P.; Meerschaert, K.; Dewil, M.; Dierick, I.; Jacobs, A.; de Vriendt, E.; Guergueltcheva, V.; *et al.* Disrupted function and axonal distribution of mutant tyrosyl-tRNA synthetase in dominant intermediate Charcot-Marie-Tooth neuropathy. *Nat. Genet.* **2006**, *38*, 197–202.
8. Lopez-Bigas, N.; Olive, M.; Rabionet, R.; Ben-David, O.; Martinez-Matos, J.A.; Bravo, O.; Banchs, I.; Volpini, V.; Gasparini, P.; Avraham, K.B.; *et al.* Connexin 31 (GJB3) is expressed in the peripheral and auditory nerves and causes neuropathy and hearing impairment. *Hum. Mol. Genet.* **2001**, *10*, 947–952.
9. Einarsdottir, E.; Carlsson, A.; Minde, J.; Toolanen, G.; Svensson, O.; Solders, G.; Holmgren, G.; Holmberg, D.; Holmberg, M. A mutation in the nerve growth factor beta gene (NGFB) causes loss of pain perception. *Hum. Mol. Genet.* **2004**, *13*, 799–805.
10. Indo, Y.; Tsuruta, M.; Hayashida, Y.; Karim, M.A.; Ohta, K.; Kawano, T.; Mitsubuchi, H.; Tonoki, H.; Awaya, Y.; Matsuda, I. Mutations in the TRKA/NGF receptor gene in patients with congenital insensitivity to pain with anhidrosis. *Nat. Genet.* **1996**, *13*, 485–488.
11. Bird, T.D.; Ott, J.; Giblett, E.R. Evidence for linkage of Charcot-Marie-Tooth neuropathy to the Duffy locus on chromosome 1. *Am. J. Hum. Genet.* **1982**, *34*, 388–394.
12. Hayasaka, K.; Himoro, M.; Sato, W.; Takada, G.; Uyemura, K.; Shimizu, N.; Bird, T.; Conneally, P.M.; Chance, P.F. Charcot-Marie-Tooth neuropathy type 1B is associated with mutations of the myelin P0 gene. *Nat. Genet.* **1993**, *5*, 31–34.
13. De Sandre-Giovannoli, A.; Chaouch, M.; Kozlov, S.; Vallat, J.M.; Tazir, M.; Kassouri, N.; Szepetowski, P.; Hammadouche, T.; Vandenberghe, A.; Stewart, C.L.; *et al.* Homozygous defects in LMNA, encoding lamin A/C nuclear-envelope proteins, cause autosomal recessive axonal neuropathy in human (Charcot-Marie-Tooth disorder type 2) and mouse. *Am. J. Hum. Genet.* **2002**, *70*, 726–736.

14. Puls, I.; Jonnakuty, C.; LaMonte, B.H.; Holzbaur, E.L.; Tokito, M.; Mann, E.; Floeter, M.K.; Bidus, K.; Drayna, D.; Oh, S.J.; *et al.* Mutant dynactin in motor neuron disease. *Nat. Genet.* **2003**, *33*, 455–456.
15. Beetz, C.; Pieber, T.R.; Hertel, N.; Schabhuttl, M.; Fischer, C.; Trajanoski, S.; Graf, E.; Keiner, S.; Kurth, I.; Wieland, T.; Varga, R.E.; *et al.* Exome sequencing identifies a REEP1 mutation involved in distal hereditary motor neuropathy type V. *Am. J. Hum. Genet.* **2012**, *91*, 139–145.
16. McEntagart, M.; Norton, N.; Williams, H.; Teare, M.D.; Dunstan, M.; Baker, P.; Houlden, H.; Reilly, M.; Wood, N.; Harper, P.S.; *et al.* Localization of the gene for distal hereditary motor neuronopathy VII (dHMN-VII) to chromosome 2q14. *Am. J. Hum. Genet.* **2001**, *68*, 1270–1276.
17. Barwick, K.E.; Wright, J.; Al-Turki, S.; McEntagart, M.M.; Nair, A.; Chioza, B.; Al-Memar, A.; Modarres, H.; Reilly, M.M.; Dick, K.J.; *et al.* Defective presynaptic choline transport underlies hereditary motor neuropathy. *Am. J. Hum. Genet.* **2012**, *91*, 1103–1107.
18. Yuan, J.; Matsuura, E.; Higuchi, Y.; Hashiguchi, A.; Nakamura, T.; Nozuma, S.; Sakiyama, Y.; Yoshimura, A.; Izumo, S.; Takashima, H. Hereditary sensory and autonomic neuropathy type IID caused by an SCN9A mutation. *Neurology* **2013**, *80*, 1641–1649.
19. Meijer, I.A.; Vanasse, M.; Nizard, S.; Robitaille, Y.; Rossignol, E. An atypical case of SCN9A mutation presenting with global motor delay and a severe pain disorder. *Muscle Nerve* **2013**, *49*, 134–138.
20. Blumen, S.C.; Astord, S.; Robin, V.; Vignaud, L.; Toumi, N.; Cieslik, A.; Achiron, A.; Carasso, R.L.; Gurevich, M.; Braverman, I.; *et al.* A rare recessive distal hereditary motor neuropathy with HSJ1 chaperone mutation. *Ann. Neurol.* **2012**, *71*, 509–519.
21. Riviere, J.B.; Ramalingam, S.; Lavastre, V.; Shekarabi, M.; Holbert, S.; Lafontaine, J.; Srour, M.; Merner, N.; Rochefort, D.; Hince, P.; *et al.* KIF1A, an axonal transporter of synaptic vesicles, is mutated in hereditary sensory and autonomic neuropathy type 2. *Am. J. Hum. Genet.* **2011**, *89*, 219–230.
22. Leipold, E.; Liebmann, L.; Korenke, G.C.; Heinrich, T.; Giesselmann, S.; Baets, J.; Ebbinghaus, M.; Goral, R.O.; Stodberg, T.; Hennings, J.C.; *et al.* A de novo gain-of-function mutation in SCN11A causes loss of pain perception. *Nat. Genet.* **2013**, *45*,1399–1404.
23. Kok, C.; Kennerson, M.L.; Spring, P.J.; Ing, A.J.; Pollard, J.D.; Nicholson, G.A. A locus for hereditary sensory neuropathy with cough and gastroesophageal reflux on chromosome 3p22-p24. *Am. J. Hum. Genet.* **2003**, *73*, 632–637.
24. Spring, P.J.; Kok, C.; Nicholson, G.A.; Ing, A.J.; Spies, J.M.; Bassett, M.L.; Cameron, J.; Kerlin, P.; Bowler, S.; Tuck, R.; *et al.* Autosomal dominant hereditary sensory neuropathy with chronic cough and gastro-oesophageal reflux: Clinical features in two families linked to chromosome 3p22-p24. *Brain* **2005**, *128*, 2797–2810.
25. Ishiura, H.; Sako, W.; Yoshida, M.; Kawarai, T.; Tanabe, O.; Goto, J.; Takahashi, Y.; Date, H.; Mitsui, J.; Ahsan, B.; *et al.* The TRK-fused gene is mutated in hereditary motor and sensory neuropathy with proximal dominant involvement. *Am. J. Hum. Genet.* **2012**, *91*, 320–329.
26. Kwon, J.M.; Elliott, J.L.; Yee, W.C.; Ivanovich, J.; Scavarda, N.J.; Moolsintong, P.J.; Goodfellow, P.J. Assignment of a second Charcot-Marie-Tooth type II locus to chromosome 3q. *Am. J. Hum. Genet.* **1995**, *57*, 853–858.

27. Verhoeven, K.; de Jonghe, P.; Coen, K.; Verpoorten, N.; Auer-Grumbach, M.; Kwon, J.M.; FitzPatrick, D.; Smedding, E.; de Vriendt, E.; Jacobs, A.; *et al.* Mutations in the small GTP-ase late endosomal protein RAB7 cause Charcot-Marie-Tooth type 2B neuropathy. *Am. J. Hum. Genet.* **2003**, *72*, 722–727.
28. Soong, B.W.; Huang, Y.H.; Tsai, P.C.; Huang, C.C.; Pan, H.C.; Lu, Y.C.; Chien, H.J.; Liu, T.T.; Chang, M.H.; Lin, K.P.; *et al.* Exome sequencing identifies GNB4 mutations as a cause of dominant intermediate Charcot-Marie-Tooth disease. *Am. J. Hum. Genet.* **2013**, *92*, 422–430.
29. Ylikallio, E.; Poyhonen, R.; Zimon, M.; de Vriendt, E.; Hilander, T.; Paetau, A.; Jordanova, A.; Lonnqvist, T.; Tyynismaa, H. Deficiency of the E3 ubiquitin ligase TRIM2 in early-onset axonal neuropathy. *Hum. Mol. Genet.* **2013**, *22*, 2975–2983.
30. Bouhouche, A.; Benomar, A.; Bouslam, N.; Ouazzani, R.; Chkili, T.; Yahyaoui, M. Autosomal recessive mutilating sensory neuropathy with spastic paraplegia maps to chromosome 5p15.31–14.1. *Eur. J. Hum. Genet.* **2006**, *14*, 249–252.
31. Bouhouche, A.; Benomar, A.; Bouslam, N.; Chkili, T.; Yahyaoui, M. Mutation in the epsilon subunit of the cytosolic chaperonin-containing t-complex peptide-1 (Cct5) gene causes autosomal recessive mutilating sensory neuropathy with spastic paraplegia. *J. Med. Genet.* **2006**, *43*, 441–443.
32. Kurth, I.; Pamminger, T.; Hennings, J.C.; Soehendra, D.; Huebner, A.K.; Rotthier, A.; Baets, J.; Senderek, J.; Topaloglu, H.; Farrell, S.A.; *et al.* Mutations in FAM134B, encoding a newly identified Golgi protein, cause severe sensory and autonomic neuropathy. *Nat. Genet.* **2009**, *41*, 1179–1181.
33. Kolb, S.J.; Snyder, P.J.; Poi, E.J.; Renard, E.A.; Bartlett, A.; Gu, S.; Sutton, S.; Arnold, W.D.; Freimer, M.L.; Lawson, V.H.; *et al.* Mutant small heat shock protein B3 causes motor neuropathy: Utility of a candidate gene approach. *Neurology* **2010**, *74*, 502–506.
34. LeGuern, E.; Guilbot, A.; Kessali, M.; Ravisé, N.; Tassin, J.; Maisonobe, T.; Grid, D.; Brice, A. Homozygosity mapping of an autosomal recessive form of demyelinating Charcot-Marie-Tooth disease to chromosome 5q23-q33. *Hum. Mol. Genet.* **1996**, *5*, 1685–1688.
35. Senderek, J.; Bergmann, C.; Stendel, C.; Kirfel, J.; Verpoorten, N.; de Jonghe, P.; Timmerman, V.; Chrast, R.; Verheijen, M.H.G.; Lemke, G.; *et al.* Mutations in a gene encoding a novel SH3/TPR domain protein cause autosomal recessive Charcot-Marie-Tooth type 4C neuropathy. *Am. J. Hum. Genet.* **2003**, *73*, 1106–1119.
36. Zimon, M.; Baets, J.; Almeida-Souza, L.; de Vriendt, E.; Nikodinovic, J.; Parman, Y.; Battalo Gcaron, L.E.; Matur, Z.; Guergueltcheva, V.; Tournev, I.; *et al.* Loss-of-function mutations in HINT1 cause axonal neuropathy with neuromyotonia. *Nat. Genet.* **2012**, *44*, 1080–1083.
37. Vester, A.; Velez-Ruiz, G.; McLaughlin, H.M.; Lupski, J.R.; Talbot, K.; Vance, J.M.; Zuchner, S.; Roda, R.H.; Fischbeck, K.H.; Biesecker, L.G.; *et al.* A loss-of-function variant in the human histidyl-tRNA synthetase (HARS) gene is neurotoxic *in vivo*. *Hum. Mutat.* **2013**, *34*, 191–199.
38. Sumner, C.J.; d'Ydewalle, C.; Wooley, J.; Fawcett, K.A.; Hernandez, D.; Gardiner, A.R.; Kalmar, B.; Baloh, R.H.; Gonzalez, M.; Zuchner, S.; *et al.* A dominant mutation in FBXO38 causes distal spinal muscular atrophy with calf predominance. *Am. J. Hum. Genet.* **2013**, *93*, 976–983.

39. Edvardson, S.; Cinnamon, Y.; Jalas, C.; Shaag, A.; Maayan, C.; Axelrod, F.B.; Elpeleg, O. Hereditary sensory autonomic neuropathy caused by a mutation in dystonin. *Ann. Neurol.* **2012**, *71*, 569–572.
40. Chow, C.Y.; Zhang, Y.L.; Dowling, J.J.; Jin, N.; Adamska, M.; Shiga, K.; Szigeti, K.; Shy, M.E.; Li, J.; Zhang, X.B.; *et al.* Mutation of FIG4 causes neurodegeneration in the pale tremor mouse and patients with CMT4J. *Nature* **2007**, *448*, 68–72.
41. Ionasescu, V.V.; Searby, C.; Sheffield, V.C.; Roklina, T.; Nishimura, D.; Ionasescu, R. Autosomal dominant Charcot-Marie-Tooth axonal neuropathy mapped on chromosome 7p (CMT2D). *Hum. Mol. Genet.* **1996**, *5*, 1373–1375.
42. Pericak-Vance, M.A.; Speer, M.C.; Lennon, F.; West, S.G.; Menold, M.M.; Stajich, J.M.; Wolpert, C.M.; Slotterbeck, B.D.; Saito, M.; Tim, R.W.; *et al.* Confirmation of a second locus for CMT2 and evidence for additional genetic heterogeneity. *Neurogenetics* **1997**, *1*, 89–93.
43. Christodoulou, K.; Kyriakides, T.; Hristova, A.H.; Georgiou, D.M.; Kalaydjieva, L.; Yshpekova, B.; Ivanova, T.; Weber, J.L.; Middleton, L.T. Mapping of a distal form of spinal muscular atrophy with upper limb predominance to chromosome 7p. *Hum. Mol. Genet.* **1995**, *4*, 1629–1632.
44. Antonellis, A.; Ellsworth, R.E.; Sambuughin, N.; Puls, I.; Abel, A.; Lee-Lin, S.Q.; Jordanova, A.; Kremensky, I.; Christodoulou, K.; Middleton, L.T.; *et al.* Glycyl tRNA synthetase mutations in Charcot-Marie-Tooth disease type 2D and distal spinal muscular atrophy type V. *Am. J. Hum. Genet.* **2003**, *72*, 1293–1299.
45. Sambuughin, N.; Sivakumar, K.; Selenge, B.; Lee, H.S.; Friedlich, D.; Baasanjav, D.; Dalakas, M.C.; Goldfarb, L.G. Autosomal dominant distal spinal muscular atrophy type V (dSMA-V) and Charcot-Marie-Tooth disease type 2D (CMT2D) segregate within a single large kindred and map to a refined region on chromosome 7p15. *J. Neurol. Sci.* **1998**, *161*, 23–28.
46. Ismailov, S.M.; Fedotov, V.P.; Dadali, E.L.; Polyakov, A.V.; van Broeckhoven, C.; Ivanov, V.I.; de Jonghe, P.; Timmerman, V.; Evgrafov, O.V. A new locus for autosomal dominant Charcot-Marie-Tooth disease type 2 (CMT2F) maps to chromosome 7q11-q21. *Eur. J. Hum. Genet.* **2001**, *9*, 646–650.
47. Evgrafov, O.V.; Mersiyanova, I.V.; Irobi, J.; van den Bosch, L.; Dierick, I.; Schagina, O.; Verpoorten, N.; van Impe, K.; Fedotov, V.P.; Dadali, E.L.; *et al.* Mutant small heat-shock protein 27 causes axonal Charcot-Marie-Tooth disease and distal hereditary motor neuropathy. *Nat. Genet.* **2004**, *36*, 602–606.
48. Brkanac, Z.; Fernandez, M.; Matsushita, M.; Lipe, H.; Wolff, J.; Bird, T.D.; Raskind, W.H. Autosomal dominant sensory/motor neuropathy with Ataxia (SMNA): Linkage to chromosome 7q22-q32. *Am. J. Med. Genet.* **2002**, *114*, 450–457.
49. Brkanac, Z.; Spencer, D.; Shendure, J.; Robertson, P.D.; Matsushita, M.; Vu, T.; Bird, T.D.; Olson, M.V.; Raskind, W.H. IFRD1 is a candidate gene for SMNA on chromosome 7q22-q23. *Am. J. Hum. Genet.* **2009**, *84*, 692–697.
50. Verhoeven, K.; de Jonghe, P.; van de Putte, T.; Nelis, E.; Zwijsen, A.; Verpoorten, N.; de Vriendt, E.; Jacobs, A.; van Gerwen, V.; Francis, A.; *et al.* Slowed conduction and thin myelination of peripheral nerves associated with mutant Rho guanine nucleotide exchange factor 10. *Am. J. Hum. Genet.* **2003**, *73*, 926–932.

51. Mersiyanova, I.V.; Perepelov, A.V.; Polyakov, A.V.; Sitnikov, V.F.; Dadali, E.L.; Oparin, R.B.; Petrin, A.; Evgrafov, O.V. A new variant of Charcot-Marie-Tooth disease type 2 (CMT2E) is probably the result of a mutation in the neurofilament light gene. *Am. J. Hum. Genet.* **2000**, *67*, 37–46.
52. Baxter, R.V.; Ben Othmane, K.; Rochelle, J.M.; Stajich, J.E.; Hulette, C.; Dew-Knight, S.; Hentati, F.; Ben Hamida, M.; Bel, S.; Stenger, J.E.; *et al.* Ganglioside-induced differentiation-associated protein-1 is mutant in Charcot-Marie-Tooth disease type 4A/8q21. *Nat. Genet.* **2002**, *30*, 21–22.
53. Ben Othmane, K.; Hentati, F.; Lennon, F.; Ben Hamida, C.; Blel, S.; Roses, A.D.; Pericak-Vance, M.A.; Ben Hamida, M.; Vance, J.M. Linkage of a locus (CMT4A) for autosomal recessive Charcot-Marie-Tooth disease to chromosome 8q. *Hum. Mol. Genet.* **1993**, *2*, 1625–1628.
54. Cuesta, A.; Pedrola, L.; Sevilla, T.; Garcia-Planells, J.; Chumillas, M.J.; Mayordomo, F.; LeGuern, E.; Marin, I.; Vilchez, J.J.; Palau, F. The gene encoding ganglioside-induced differentiation-associated protein 1 is mutated in axonal Charcot-Marie-Tooth type 4A disease. *Nat. Genet.* **2002**, *30*, 22–25.
55. Kalaydjieva, L.; Hallmayer, J.; Chandler, D.; Savov, A.; Nikolova, A.; Angelicheva, D.; King, R.H.; Ishpekova, B.; Honeyman, K.; Calafell, F.; *et al.* Gene mapping in Gypsies identifies a novel demyelinating neuropathy on chromosome 8q24. *Nat. Genet.* **1996**, *14*, 214–217.
56. Kalaydjieva, L.; Gresham, D.; Gooding, R.; Heather, L.; Baas, F.; de Jonge, R.; Blechschmidt, K.; Angelicheva, D.; Chandler, D.; Worsley, P.; *et al.* N-myc downstream-regulated gene 1 is mutated in hereditary motor and sensory neuropathy—Lom. *Am. J. Hum. Genet.* **2000**, *67*, 47–58.
57. Ionasescu, V.V.; Kimura, J.; Searby, C.C.; Smith, W.L., Jr.; Ross, M.A.; Ionasescu, R. A Déjérine-Sottas neuropathy family with a gene mapped on chromosome 8. *Muscle Nerve* **1996**, *19*, 319–323.
58. Christodoulou, K.; Zamba, E.; Tsingis, M.; Mubaidin, A.; Horany, K.; Abu-Sheikh, S.; El-Khateeb, M.; Kyriacou, K.; Kyriakides, T.; Al-Qudah, A.; *et al.* A novel form of distal hereditary motor neuronopathy maps to chromosome 9p21.1-p12. *Ann. Neurol.* **2000**, *48*, 877–884.
59. Middleton, L.T.; Christodoulou, K.; Mubaidin, A.; Zamba, E.; Tsingis, M.; Kyriacou, K.; Abu-Sheikh, S.; Kyriakides, T.; Neocleous, V.; Georgiou, D.M.; *et al.* Distal hereditary motor neuronopathy of the Jerash type. *Ann. N. Y. Acad. Sci.* **1999**, *883*, 65–68.
60. Oates, E.C.; Rossor, A.M.; Hafezparast, M.; Gonzalez, M.; Speziani, F.; Macarthur, D.G.; Lek, M.; Cottenie, E.; Scoto, M.; Foley, A.R.; *et al.* Mutations in BICD2 Cause Dominant Congenital Spinal Muscular Atrophy and Hereditary Spastic Paraplegia. *Am. J. Hum. Genet.* **2013**, *92*, 965–973.
61. Peeters, K.; Litvinenko, I.; Asselbergh, B.; Almeida-Souza, L.; Chamova, T.; Geuens, T.; Ydens, E.; Zimon, M.; Irobi, J.; de Vriendt, E.; *et al.* Molecular defects in the motor adaptor BICD2 cause proximal spinal muscular atrophy with autosomal-dominant inheritance. *Am. J. Hum. Genet.* **2013**, *92*, 955–964.

62. Neveling, K.; Martinez-Carrera, L.A.; Holker, I.; Heister, A.; Verrips, A.; Hosseini-Barkooie, S.M.; Gilissen, C.; Vermeer, S.; Pennings, M.; Meijer, R.; *et al.* Mutations in BICD2, which encodes a golgin and important motor adaptor, cause congenital autosomal-dominant spinal muscular atrophy. *Am. J. Hum. Genet.* **2013**, *92*, 946–954.
63. Blair, I.P.; Hulme, D.; Dawkins, J.L.; Nicholson, G.A. A YAC-based transcript map of human chromosome 9q22.1-q22.3 encompassing the loci for hereditary sensory neuropathy type I and multiple self-healing squamous epithelioma. *Genomics* **1998**, *51*, 277–281.
64. Blair, I.P.; Dawkins, J.L.; Nicholson, G.A. Fine mapping of the hereditary sensory neuropathy type I locus on chromosome 9q22.1-->q22.3: Exclusion of GAS1 and XPA. *Cytogenet. Cell Genet.* **1997**, *78*, 140–144.
65. Dawkins, J.L.; Hulme, D.J.; Brahmbhatt, S.B.; Auer-Grumbach, M.; Nicholson, G.A. Mutations in SPTLC1, encoding serine palmitoyltransferase, long chain base subunit-1, cause hereditary sensory neuropathy type I. *Nat. Genet.* **2001**, *27*, 309–312.
66. Nicholson, G.A.; Dawkins, J.L.; Blair, I.P.; Kennerson, M.L.; Gordon, M.J.; Cherryson, A.K.; Nash, A.; Bananis, T. The gene for hereditary sensory neuropathy type I (HSN-I) maps to chromosome 9q22.1-q22.3. *Nat. Genet.* **1996**, *13*, 101–104.
67. Bejaoui, K.; Wu, C.; Scheffler, M.D.; Haan, G.; Ashby, P.; Wu, L.; de Jong, P.; Brown, R.H., Jr. SPTLC1 is mutated in hereditary sensory neuropathy, type 1. *Nat. Genet.* **2001**, *27*, 261–262.
68. Blumenfeld, A.; Slaugenhaupt, S.A.; Axelrod, F.B.; Lucente, D.E.; Maayan, C.; Liebert, C.B.; Ozelius, L.J.; Trofatter, J.A.; Haines, J.L.; Breakefield, X.O.; *et al.* Localization of the gene for familial dysautonomia on chromosome 9 and definition of DNA markers for genetic diagnosis. *Nat. Genet.* **1993**, *4*, 160–164.
69. Slaugenhaupt, S.A.; Blumenfeld, A.; Gill, S.P.; Leyne, M.; Mull, J.; Cuajungco, M.P.; Liebert, C.B.; Chadwick, B.; Idelson, M.; Reznik, L.; *et al.* Tissue-specific expression of a splicing mutation in the IKBKAP gene causes familial dysautonomia. *Am. J. Hum. Genet.* **2001**, *68*, 598–605.
70. Anderson, S.L.; Coli, R.; Daly, I.W.; Kichula, E.A.; Rork, M.J.; Volpi, S.A.; Ekstein, J.; Rubin, B.Y. Familial dysautonomia is caused by mutations of the IKAP gene. *Am. J. Hum. Genet.* **2001**, *68*, 753–758.
71. Guernsey, D.L.; Jiang, H.; Bedard, K.; Evans, S.C.; Ferguson, M.; Matsuoka, M.; Macgillivray, C.; Nightingale, M.; Perry, S.; Rideout, A.L.; *et al.* Mutation in the gene encoding ubiquitin ligase LRSAM1 in patients with Charcot-Marie-Tooth disease. *PLoS Genet.* **2010**, *6*, doi:10.1371/journal.pgen.1001081.
72. Weterman, M.A.; Sorrentino, V.; Kasher, P.R.; Jakobs, M.E.; van Engelen, B.G.; Fluiter, K.; de Wissel, M.B.; Sizarov, A.; Nurnberg, G.; Nurnberg, P.; *et al.* A frameshift mutation in LRSAM1 is responsible for a dominant hereditary polyneuropathy. *Hum. Mol. Genet.* **2012**, *21*, 358–370.
73. Blair, I.P.; Bennett, C.L.; Abel, A.; Rabin, B.A.; Griffin, J.W.; Fischbeck, K.H.; Cornblath, D.R.; Chance, P.F. A gene for autosomal dominant juvenile amyotrophic lateral sclerosis (ALS4) localizes to a 500-kb interval on chromosome 9q34. *Neurogenetics* **2000**, *3*, 1–6.
74. Chance, P.F.; Rabin, B.A.; Ryan, S.G.; Ding, Y.; Scavina, M.; Crain, B.J.; Griffin, J.W.; Cornblath, D.R. Linkage to the gene for an autosomal dominant form of juvenile amyotrophic lateral sclerosis to chromosome 9q34. *Am. J. Hum. Genet.* **1998**, *62*, 640.

75. Chen, Y.-Z.; Bennett, C.L.; Huynh, H.M.; Blair, I.P.; Puls, I.; Irobi, J.; Dierick, I.; Abel, A.; Kennerson, M.L.; Rabin, B.A.; *et al.* DNA/RNA helicase gene mutations in a form of juvenile Amyotrophic Lateral Sclerosis (ALS4). *Am. J. Hum. Genet.* **2004**, *74*, 1128–1135.
76. Echaniz-Laguna, A.; Ghezzi, D.; Chassagne, M.; Mayencon, M.; Padet, S.; Melchionda, L.; Rouvet, I.; Lannes, B.; Bozon, D.; Latour, P.; *et al.* SURF1 deficiency causes demyelinating Charcot-Marie-Tooth disease. *Neurology* **2013**, *81*, 1523–1530.
77. Xu, W.Y.; Gu, M.M.; Sun, L.H.; Guo, W.T.; Zhu, H.B.; Ma, J.F.; Yuan, W.T.; Kuang, Y.; Ji, B.J.; Wu, X.L.; *et al.* A nonsense mutation in DHTKD1 causes Charcot-Marie-Tooth disease type 2 in a large Chinese pedigree. *Am. J. Hum. Genet.* **2012**, *91*, 1088–1094.
78. Warner, L.E.; Mancias, P.; Butler, I.J.; McDonald, C.M.; Keppen, L.; Koob, G.; Lupski, J.R. Mutations in the early growth response 2 (*EGR2*) gene are associated with hereditary myelinopathies. *Nat. Genet.* **1998**, *18*, 382–384.
79. Hantke, J.; Rogers, T.; French, L.; Tournev, I.; Guergueltcheva, V.; Urtizberea, J.A.; Colomer, J.; Corches, A.; Lupu, C.; Merlini, L.; *et al.* Refined mapping of the HMSNR critical gene region–construction of a high-density integrated genetic and physical map. *Neuromuscul. Disord.* **2003**, *13*, 729–736.
80. Rogers, T.; Chandler, D.; Angelicheva, D.; Thomas, P.K.; Youl, B.; Tournev, I.; Gergelcheva, V.; Kalaydjieva, L. A novel locus for autosomal recessive peripheral neuropathy in the EGR2 region on 10q23. *Am. J. Hum. Genet.* **2000**, *67*, 664–671.
81. Hantke, J.; Chandler, D.; King, R.; Wanders, R.J.; Angelicheva, D.; Tournev, I.; McNamara, E.; Kwa, M.; Guergueltcheva, V.; Kaneva, R.; *et al.* A mutation in an alternative untranslated exon of hexokinase 1 associated with hereditary motor and sensory neuropathy—Russe (HMSNR). *Eur. J. Hum. Genet.* **2009**, *17*, 1606–1614.
82. Verhoeven, K.; Villanova, M.; Rossi, A.; Malandrini, A.; de Jonghe, P.; Timmerman, V. Localization of the gene for the intermediate form of Charcot-Marie-Tooth to chromosome 10q24.1-q25.1. *Am. J. Hum. Genet.* **2001**, *69*, 889–894.
83. Villanova, M.; Timmerman, V.; de Jonghe, P.; Rizzuto, N.; van Broeckhoven, C.; Guazzi, G.; Rossi, A. Charcot-Marie-Tooth disease: An intermediate form. *Neuromusc. Disord.* **1998**, *8*, 392–393.
84. Gambardella, A.; Bolino, A.; Muglia, M.; Bono, F.; Valentino, P.; Oliveri, R.L.; Sabatelli, M.; Brancolini, C.; van Broeckhoven, C.; Romeo, G.; *et al.* Genetic heterogeneity in autosomal recessive hereditary motor and sensory neuropathy with focally folded myelin sheats (CMT4B). *Neurology* **1998**, *50*, 799–801.
85. Ben Othmane, K.; Johnson, E.; Menold, M.; Graham, F.L.; Hamida, M.B.; Hasegawa, O.; Rogala, A.D.; Ohnishi, A.; Pericak-Vance, M.; Hentati, F.; *et al.* Identification of a new locus for autosomal recessive Charcot-Marie-Tooth disease with focally folded myelin on chromosome 11p15. *Genomics* **1999**, *62*, 344–349.
86. Azzedine, H.; Bolino, A.; Taieb, T.; Birouk, N.; di Duca, M.; Bouhouche, A.; Benamou, S.; Mrabet, A.; Hammadouche, T.; Chkili, T.; *et al.* Mutations in MTMR13, a new pseudophosphatase homologue of MTMR2 and Sbf1, in two families with an autosomal recessive demyelinating form of Charcot-Marie-Tooth disease associated with early-onset glaucoma. *Am. J. Hum. Genet.* **2003**, *72*, 1141–1153.

87. Kiwaki, T.; Umehara, F.; Takashima, H.; Nakagawa, M.; Kamimura, K.; Kashio, N.; Sakamoto, Y.; Unoki, K.; Nobuhara, Y.; Michizono, K.; *et al.* Hereditary motor and sensory neuropathy with myelin folding and juvenile onset glaucoma. *Neurology* **2000**, *55*, 392–397.
88. Auer-Grumbach, M.; Loscher, W.N.; Wagner, K.; Petek, E.; Korner, E.; Offenbacher, H.; Hartung, H.P. Phenotypic and genotypic heterogeneity in hereditary motor neuronopathy type V: A clinical, electrophysiological and genetic study. *Brain* **2000**, *123*, 1612–1623.
89. Windpassinger, C.; Auer-Grumbach, M.; Irobi, J.; Patel, H.; Petek, E.; Hörl, G.; Malli, R.; Dierick, I.; Warner, T.; Proukakis, C.; *et al.* Heterozygous missense mutations in the *BSCL2* are associated with distal hereditary motor neuropathy and Silver syndrome. *Nat. Genet.* **2004**, *36*, 271–276.
90. Windpassinger, C.; Wagner, K.; Petek, E.; Fischer, R.; Auer-Grumbach, M. Refinement of the "Silver syndrome locus" on chromosome 11q12-q14 in four families and exclusion of eight candidate genes. *Hum. Genet.* **2003,** *114*, 99–109.
91. Grohmann, K.; Wienker, T.F.; Saar, K.; Rudnik-Schoneborn, S.; Stoltenburg-Didinger, G.; Rossi, R.; Novelli, G.; Nurnberg, G.; Pfeufer, A.; Wirth, B.; *et al.* Diaphragmatic spinal muscular atrophy with respiratory distress is heterogeneous, and one form is linked to chromosome 11q13-q21. *Am. J. Hum. Genet.* **1999**, *65*, 1459–1462.
92. Grohmann, K.; Schuelke, M.; Diers, A.; Hoffmann, K.; Lucke, B.; Adams, C.; Bertini, E.; Leonhardt-Horti, H.; Muntoni, F.; Ouvrier, R.; *et al.* Mutations in the gene encoding immunoglobulin μ-binding protein 2 cause spinal muscular atrophy with respiratory distress type 1. *Nat. Genet.* **2001**, *29*, 75–77.
93. Bolino, A.; Brancolini, V.; Bono, F.; Bruni, A.; Gambardella, A.; Romeo, G.; Quattrone, A.; Devoto, M. Localization of a gene responsible for autosomal recessive demyelinating neuropathy with focally folded myelin sheaths to chromosome 11q23 by homozygosity mapping and haplotype sharing. *Hum. Mol. Genet.* **1996**, *5*, 1051–1054.
94. Bolino, A.; Muglia, M.; Conforti, F.L.; LeGuern, E.; Salih, M.A.; Georgiou, D.M.; Christodoulou, K.; Hausmanowa-Petrusewicz, I.; Mandich, P.; Schenone, A.; *et al.* Charcot-Marie-Tooth type 4B is caused by mutations in the gene encoding myotubularin-related protein-2. *Nat. Genet.* **2000**, *25*, 17–19.
95. Bolino, A.; Levy, E.R.; Muglia, M.; Conforti, F.L.; LeGuern, E.; Salih, M.A.; Georgiou, D.M.; Christodoulou, R.K.; Hausmanowa-Petrusewicz, I.; Mandich, P.; *et al.* Genetic refinement and physical mapping of the CMT4B gene on chromosome 11q22. *Genomics* **2000**, *63*, 271–278.
96. Quattrone, A.; Gambardella, A.; Bono, F.; Aguglia, U.; Bolino, A.; Bruni, A.C.; Montesi, M.P.; Oliveri, R.L.; Sabatelli, M.; Tamburrini, O.; *et al.* Autosomal recessive hereditary motor and sensory neuropathy with focally folded myelin sheaths: Clinical, electrophysiologic, and genetic aspects of a large family. *Neurology* **1996**, *46*, 1318–1324.
97. Lafrenière, R.G.; MacDonald, M.L.; Dube, M.P.; MacFarlane, J.; O'Driscoll, M.; Brais, B.; Meilleur, S.; Brinkman, R.R.; Dadivas, O.; Pape, T.; *et al.* Identification of a novel gene (HSN2) causing hereditary sensory and autonomic neuropathy type II through the Study of Canadian Genetic Isolates. *Am. J. Hum. Genet.* **2004**, *74*, 1064–1073.

98. De Sandre-Giovannoli, A.; Delague, V.; Hamadouche, T.; Chaouch, M.; Krahn, M.; Boccaccio, I.; Maisonobe, T.; Chouery, E.; Jabbour, R.; Atweh, S.; *et al.* Homozygosity mapping of autosomal recessive demyelinating Charcot-Marie-Tooth neuropathy (CMT4H) to a novel locus on chromosome 12p11.21-q13.11. *J. Med. Genet.* **2005**, *42*, 260–265.
99. Stendel, C.; Roos, A.; Deconinck, T.; Pereira, J.; Castagner, F.; Niemann, A.; Kirschner, J.; Korinthenberg, R.; Ketelsen, U.P.; Battaloglu, E.; *et al.* Peripheral nerve demyelination caused by a mutant Rho GTPase guanine nucleotide exchange factor, frabin/FGD4. *Am. J. Hum. Genet.* **2007**, *81,* 158–164.
100. Delague, V.; Jacquier, A.; Hamadouche, T.; Poitelon, Y.; Baudot, C.; Boccaccio, I.; Chouery, E.; Chaouch, M.; Kassouri, N.; Jabbour, R.; *et al.* Mutations in FGD4 encoding the Rho GDP/GTP exchange factor FRABIN cause autosomal recessive Charcot-Marie-Tooth type 4H. *Am. J. Hum. Genet.* **2007**, *81*, 1–16.
101. Nelis, E.; Berciano, J.; Verpoorten, N.; Coen, K.; Dierick, I.; van Gerwen, V.; Combarros, O.; de Jonghe, P.; Timmerman, V. Autosomal dominant axonal Charcot-Marie-Tooth disease type 2 (CMT2G) maps to chromosome 12q12-q13.3. *J. Med. Genet.* **2004**, *41*, 193–197.
102. Dyck, P.J.; Litchy, W.J.; Minnerath, S.; Bird, T.D.; Chance, P.F.; Schiad, D.J.; Aronson, A.E. Hereditary motor and sensory neuropathy with diaphragm and vocal cord paresis. *Ann. Neurol.* **1994**, *35*, 608–615.
103. Klein, C.J.; Cunningham, J.M.; Atkinson, E.J.; Schaid, D.J.; Hebbring, S.J.; Anderson, S.A.; Klein, D.M.; Dyck, P.J.; Litchy, W.J.; Thibodeau, S.N.; *et al.* The gene for HMSN2C maps to 12q23–24: A region of neuromuscular disorders. *Neurology* **2003**, *60*, 1151–1156.
104. McEntagart, M.E.; Reid, S.L.; Irrthum, A.; Douglas, J.B.; Eyre, K.E.; Donaghy, M.J.; Anderson, N.E.; Rahman, N. Confirmation of a hereditary motor and sensory neuropathy IIC locus at chromosome 12q23-q24. *Ann. Neurol.* **2005**, *57*, 293–297.
105. Landoure, G.; Zdebik, A.A.; Martinez, T.L.; Burnett, B.G.; Stanescu, H.C.; Inada, H.; Shi, Y.; Taye, A.A.; Kong, L.; Munns, C.H.; *et al.* Mutations in TRPV4 cause Charcot-Marie-Tooth disease type 2C. *Nat. Genet.* **2010**, *42*, 170–174.
106. Irobi, J.; Tissir, F.; de Jonghe, P.; de Vriendt, E.; van Broeckhoven, C.; Timmerman, V.; Beuten, J. A clone contig of 12q24.3 encompassing the distal hereditary motor neuropathy type II gene. *Genomics* **2000**, *65*, 34–43.
107. Irobi, J.; van Impe, K.; Seeman, P.; Jordanova, A.; Dierick, I.; Verpoorten, N.; Michalik, A.; de Vriendt, E.; Jacobs, A.; van Gerwen, V.; *et al.* Hot-spot residue in small heat-shock protein 22 causes distal motor neuropathy. *Nat. Genet.* **2004**, *36*, 597–601.
108. Timmerman, V.; de Jonghe, P.; Simokovic, S.; Löfgren, A.; Beuten, J.; Nelis, E.; Ceuterick, C.; Martin, J.-J.; van Broeckhoven, C. Distal hereditary motor neuropathy type II (distal HMN II): Mapping of a locus to chromosome 12q24. *Hum. Mol. Genet.* **1996**, *5*, 1065–1069.
109. Tang, B.S.; Zhao, G.H.; Luo, W.; Xia, K.; Cai, F.; Pan, Q.; Zhang, R.X.; Zhang, F.F.; Liu, X.M.; Chen, B.; *et al.* Small heat-shock protein 22 mutated in autosomal dominant Charcot-Marie-Tooth disease type 2L. *Hum. Genet.* **2005**, *116*, 222–224.
110. Tang, B.S.; Luo, W.; Xia, K.; Xiao, J.F.; Jiang, H.; Shen, L.; Tang, J.G.; Zhao, G.H.; Cai, F.; Pan, Q.; *et al.* A new locus for autosomal dominant Charcot-Marie-Tooth disease type 2 (CMT2L) maps to chromosome 12q24. *Hum. Genet.* **2004**, *114*, 527–533.

111. Guelly, C.; Zhu, P.P.; Leonardis, L.; Papic, L.; Zidar, J.; Schabhuttl, M.; Strohmaier, H.; Weis, J.; Strom, T.M.; Baets, J.; *et al.* Targeted high-throughput sequencing identifies mutations in atlastin-1 as a cause of hereditary sensory neuropathy type I. *Am. J. Hum. Genet.* **2011**, *88*, 99–105.
112. Rotthier, A.; Auer-Grumbach, M.; Janssens, K.; Baets, J.; Penno, A.; Almeida-Souza, L.; van Hoof, K.; Jacobs, A.; de Vriendt, E.; Schlotter-Weigel, B.; *et al.* Mutations in the SPTLC2 subunit of serine palmitoyltransferase cause hereditary sensory and autonomic neuropathy type I. *Am. J. Hum. Genet.* **2010**, *87*, 513–522.
113. Auer-Grumbach, M.; Weger, M.; Fink-Puches, R.; Papic, L.; Frohlich, E.; Auer-Grumbach, P.; El Shabrawi-Caelen, L.; Schabhuttl, M.; Windpassinger, C.; Senderek, J.; *et al.* Fibulin-5 mutations link inherited neuropathies, age-related macular degeneration and hyperelastic skin. *Brain* **2011**, *134*, 1839–1852.
114. Boyer, O.; Nevo, F.; Plaisier, E.; Funalot, B.; Gribouval, O.; Benoit, G.; Cong, E.H.; Arrondel, C.; Tete, M.J.; Montjean, R.; *et al.* INF2 mutations in Charcot-Marie-Tooth disease with glomerulopathy. *N. Engl. J. Med.* **2011**, *365*, 2377–2388.
115. Weedon, M.N.; Hastings, R.; Caswell, R.; Xie, W.; Paszkiewicz, K.; Antoniadi, T.; Williams, M.; King, C.; Greenhalgh, L.; Newbury-Ecob, R.; *et al.* Exome sequencing identifies a DYNC1H1 mutation in a large pedigree with dominant axonal Charcot-Marie-Tooth disease. *Am. J. Hum. Genet.* **2011**, *89*, 308–312.
116. Casaubon, L.K.; Melanson, M.; Lopes-Cendes, I.; Marineau, C.; Andermann, E.; Andermann, F.; Weissenbach, J.; Prevost, C.; Bouchard, J.P.; Mathieu, J.; *et al.* The gene responsible for a severe form of peripheral neuropathy and agenesis of the corpus callosum maps to chromosome 15q [see comments]. *Am. J. Hum. Genet.* **1996**, *58*, 28–34.
117. Howard, H.C.; Mount, D.B.; Rochefort, D.; Byun, N.; Dupre, N.; Lu, J.; Fan, X.; Song, L.; Riviere, J.B.; Prevost, C.; *et al.* The K-Cl cotransporter KCC3 is mutant in a severe peripheral neuropathy associated with agenesis of the corpus callosum. *Nat. Genet.* **2002**, *32*, 384–392.
118. Street, V.A.; Goldy, J.D.; Golden, A.S.; Tempel, B.L.; Bird, T.D.; Chance, P.F. Mapping of Charcot-Marie-Tooth disease type 1C to chromosome 16p identifies a novel locus for demyelinating neuropathies. *Am. J. Hum. Genet.* **2001**, *70*, 244–250.
119. Street, V.A.; Bennett, C.L.; Goldy, J.D.; Shirk, A.J.; Kleopa, K.A.; Tempel, B.L.; Lipe, H.P.; Scherer, S.S.; Bird, T.D.; Chance, P.F. Mutation of a putative protein degradation gene LITAF/SIMPLE in Charcot- Marie-Tooth disease 1C. *Neurology* **2003**, *60*, 22–26.
120. Latour, P.; Thauvin-Robinet, C.; Baudelet-Mery, C.; Soichot, P.; Cusin, V.; Faivre, L.; Locatelli, M.C.; Mayencon, M.; Sarcey, A.; Broussolle, E.; *et al.* A major determinant for binding and aminoacylation of tRNA(Ala) in cytoplasmic alanyl-tRNA synthetase is mutated in dominant axonal charcot-marie-tooth disease. *Am. J. Hum. Genet.* **2009**, *86*, 77–82.
121. McLaughlin, H.M.; Sakaguchi, R.; Liu, C.; Igarashi, T.; Pehlivan, D.; Chu, K.; Iyer, R.; Cruz, P.; Cherukuri, P.F.; Hansen, N.F.; *et al.* Compound heterozygosity for loss-of-function lysyl-tRNA synthetase mutations in a patient with peripheral neuropathy. *Am. J. Hum. Genet.* **2010**, *87*, 560–566.
122. Ben Hamida, C.; Cavalier, L.; Belal, S.; Sanhaji, H.; Nadal, N.; Barhoumi, C.; M’Rissa, N.; Marzouki, N.; Mandel, J.L.; Ben Hamida, M.; *et al.* Homozygosity mapping of giant axonal neuropathy gene to chromosome 16q24.1. *Neurogenetics* **1997**, *1*, 129–133.

123. Bomont, P.; Cavalier, L.; Blondeau, F.; Ben Hamida, C.; Belal, S.; Tazir, M.; Demir, E.; Korinthenberg, R.; Yalcinkaya, C.; Hentati, F.; *et al.* The gene mutated in giant axonal neuropathy encodes for gigaxonin, a novel member of the cytoskeletal BTB/Kelch repeat family. *Nat. Genet.* **2000**, *26*, 370–374.
124. Cavalier, L.; Ben Hamida, C.; Amouri, R.; Belal, S.; Bomont, P.; Lagarde, N.; Gressin, L.; Callen, D.; Demir, E.; Topaloglu, H.; *et al.* Giant axonal neuropathy locus refinement to a < 590 kb critical interval. *Eur. J. Hum. Genet.* **2000**, *8*, 527–534.
125. Doherty, E.J.; Macy, M.E.; Wang, S.M.; Dykeman, C.P.; Melanson, M.T.; Engle, E.C. CFEOM3: A new extraocular congenital fibrosis syndrome that maps to 16q24.2-q24.3. *Invest. Ophthalmol. Vis. Sci.* **1999**, *40*, 1687–1694.
126. Mackey, D.A.; Chan, W.M.; Chan, C.; Gillies, W.E.; Brooks, A.M.; O'Day, J.; Engle, E.C. Congenital fibrosis of the vertically acting extraocular muscles maps to the FEOM3 locus. *Hum. Genet.* **2002**, *110*, 510–512.
127. Tischfield, M.A.; Baris, H.N.; Wu, C.; Rudolph, G.; van Maldergem, L.; He, W.; Chan, W.M.; Andrews, C.; Demer, J.L.; Robertson, R.L.; *et al.* Human TUBB3 mutations perturb microtubule dynamics, kinesin interactions, and axon guidance. *Cell* **2010**, *140*, 74–87.
128. Vance, J.M.; Nicholson, G.A.; Yamaoka, L.H.; Stajich, J.; Stewart, J.S.; Speer, M.C.; Hung, W.-J.; Roses, A.D.; Barker, D.; Pericak-Vance, M.A. Linkage of Charcot-Marie-Tooth neuropathy type 1a to chromosome 17. *Exp. Neurol.* **1989**, *104*, 186–189.
129. Raeymaekers, P.; Timmerman, V.; Nelis, E.; de Jonghe, P.; Hoogendijk, J.E.; Baas, F.; Barker, D.F.; Martin, J.-J.; de Visser, M.; Bolhuis, P.A.; *et al.* Duplication in chromosome 17p11.2 in Charcot-Marie-Tooth neuropathy type 1a (CMT 1a). The HMSN Collaborative Research Group. *Neuromusc. Disord.* **1991**, *1*, 93–97.
130. Timmerman, V.; Nelis, E.; van Hul, W.; Nieuwenhuijsen, B.W.; Chen, K.L.; Wang, S.; Ben Othman, K.; Cullen, B.; Leach, R.J.; Hanemann, C.O.; *et al.* The peripheral myelin protein gene *PMP-22* is contained within the Charcot-Marie-Tooth disease type 1A duplication. *Nat. Genet.* **1992**, *1*, 171–175.
131. Lupski, J.R.; Wise, C.A.; Kuwano, A.; Pentao, L.; Parker, J.; Glaze, D.; Ledbetter, D.; Greenberg, F.; Patel, P.I. Gene dosage is a mechanism for Charcot-Marie-Tooth disease type 1A. *Nat. Genet.* **1992**, *1*, 29–33.
132. Patel, P.I.; Roa, B.B.; Welcher, A.A.; Schoener-Scott, R.; Trask, B.J.; Pentao, L.; Snipes, G.J.; Garcia, C.A.; Francke, U.; Shooter, E.M.; *et al.* The gene for the peripheral myelin protein PMP-22 is a candidate for Charcot-Marie-Tooth disease type 1A. *Nat. Genet.* **1992**, *1*, 159–165.
133. Chance, P.F.; Alderson, M.K.; Leppig, K.A.; Lensch, M.W.; Matsunami, N.; Smith, B.; Swanson, P.D.; Odelberg, S.J.; Distsche, C.M.; Bird, T.D. DNA deletion associated with hereditary neuropathy with liability to pressure palsies. *Cell* **1993**, *72*, 143–151.
134. Matsunami, N.; Smith, B.; Ballard, L.; Lensch, M.W.; Robertson, M.; Albertsen, H.; Hanemann, C.O.; Müller, H.W.; Bird, T.D.; White, R.; *et al.* Peripheral myelin protein-22 gene maps in the duplication in chromosome 17p11.2 associated with Charcot-Marie-Tooth 1A. *Nat. Genet.* **1992**, *1*, 176–179.

135. Valentijn, L.J.; Bolhuis, P.A.; Zorn, I.; Hoogendijk, J.E.; van den Bosch, N.; Hensels, G.W.; Stanton, V., Jr.; Housman, D.E.; Fischbeck, K.H.; Ross, D.A.; *et al.* The peripheral myelin gene *PMP-22/GAS-3* is duplicated in Charcot- Marie-Tooth disease type 1A. *Nat. Genet.* **1992**, *1*, 166–170.
136. Valentijn, L.J.; Baas, F.; Wolterman, R.A.; Hoogendijk, J.E.; van den Bosch, N.H.; Zorn, I.; Gabreëls-Festen, A.W.; de Visser, M.; Bolhuis, P.A. Identical point mutations of PMP-22 in Trembler-J mouse and Charcot-Marie-Tooth disease type 1A. *Nat. Genet.* **1992**, *2*, 288–291.
137. Pellegrino, J.E.; Rebbeck, T.R.; Brown, M.J.; Bird, T.D.; Chance, P.F. Mapping of hereditary neuralgic amyotrophy (familial brachial plexus neuropathy) to distal chromosome 17q. *Neurology* **1996**, *46*, 1128–1132.
138. Pellegrino, J.E.; George, R.A.; Biegel, J.; Farlow, M.R.; Gardner, K.; Caress, J.; Brown, M.J.; Rebbeck, T.R.; Bird, T.D.; Chance, P.F. Hereditary neuralgic amyotrophy: Evidence for genetic homogeneity and mapping to chromosome 17q25. *Hum. Genet.* **1997**, *101*, 277–283.
139. Kuhlenbaumer, G.; Hannibal, M.C.; Nelis, E.; Schirmacher, A.; Verpoorten, N.; Meuleman, J.; Watts, G.D.; Vriendt, E.D.; Young, P.; Stogbauer, F.; *et al.* Mutations in SEPT9 cause hereditary neuralgic amyotrophy. *Nat. Genet.* **2005**, *37*, 1044–1046.
140. Meuleman, J.; Kuhlenbäumer, G.; Schirmacher, A.; Wehnert, A.; de Jonghe, P.; de Vriendt, E.; Young, P.; Airaksinen, E.; Pou-Serradell, A.; Prats, J.-M.; *et al.* Genetic refinement of the hereditary neuralgic amyotrophy (HNA) locus at chromosome 17q25. *Eur. J. Hum. Genet.* **1999**, *7*, 920–927.
141. Angelicheva, D.; Turnev, I.; Dye, D.; Chandler, D.; Thomas, P.K.; Kalaydjieva, L. Congenital cataracts facial dysmorphism neuropathy (CCFDN) syndrome: A novel developmental disorder in Gypsies maps to 18qter. *Eur. J. Hum. Genet.* **1999**, *7*, 560–566.
142. Varon, R.; Gooding, R.; Steglich, C.; Marns, L.; Tang, H.; Angelicheva, D.; Yong, K.K.; Ambrugger, P.; Reinhold, A.; Morar, B.; *et al.* Partial deficiency of the C-terminal-domain phosphatase of RNA polymerase II is associated with congenital cataracts facial dysmorphism neuropathy syndrome. *Nat. Genet.* **2003**, *35*, 185–189.
143. Klein, C.J.; Botuyan, M.V.; Wu, Y.; Ward, C.J.; Nicholson, G.A.; Hammans, S.; Hojo, K.; Yamanishi, H.; Karpf, A.R.; Wallace, D.C.; *et al.* Mutations in DNMT1 cause hereditary sensory neuropathy with dementia and hearing loss. *Nat. Genet.* **2011**, *43*, 595–600.
144. Kennerson, M.L.; Zhu, D.; Gardner, R.J.; Storey, E.; Merory, J.; Robertson, S.P.; Nicholson, G.A. Dominant intermediate charcot-marie-tooth neuropathy maps to chromosome 19p12-p13.2. *Am. J. Hum. Genet.* **2001**, *69*, 883–888.
145. Zhu, D.Q.; Kennerson, M.; Merory, J.; Chrast, R.; Verheijen, M.; Lemke, G.; Nicholson, G. Refined localization of dominant intermediate Charcot-Marie-Tooth neuropathy and exclusion of seven known candidate genes in the region. *Neurogenetics* **2003**, *4*, 179–183.
146. Zuchner, S.; Noureddine, M.; Kennerson, M.; Verhoeven, K.; Claeys, K.; de Jonghe, P.; Merory, J.; Oliveira, S.A.; Speer, M.C.; Stenger, J.E.; *et al.* Mutations in the pleckstrin homology domain of dynamin 2 cause dominant intermediate Charcot-Marie-Tooth disease. *Nat. Genet.* **2005**, *37*, 289–294.

147. Speer, M.C.; Graham, F.L.; Bonner, E.; Collier, K.; Stajich, J.M.; Gaskell, P.C.; Pericak-Vance, M.A.; Vance, J.M. Reduction in the minimum candidate interval in the dominant-intermediate form of Charcot-Marie-Tooth neuropathy to D19S586 to D19S432. *Neurogenetics* **2002**, *4*, 83–85.
148. Delague, V.; Bareil, C.; Tuffery, S.; Bouvagnet, P.; Chouery, E.; Koussa, S.; Maisonobe, T.; Loiselet, J.; Megarbane, A.; Claustres, M. Mapping of a new locus for autosomal recessive demyelinating Charcot-Marie-Tooth disease to 19q13.1–13.3 in a large consanguineous Lebanese family: Exclusion of MAG as a candidate gene. *Am. J. Hum. Genet.* **2000**, *67*, 236–243.
149. Guilbot, A.; Williams, A.; Ravisé, N.; Verny, C.; Brice, A.; Sherman, D.L.; Brophy, P.J.; LeGuern, E.; Delague, V.; Bareil, C.; *et al.* A mutation in periaxin is responsible for CMT4F, an autosomal recessive form of Charcot-Marie-Tooth disease. *Hum. Mol. Genet.* **2001**, *10*, 415–421.
150. Boerkoel, C.F.; Takashima, H.; Stankiewicz, P.; Garcia, C.A.; Leber, S.M.; Rhee-Morris, L.; Lupski, J.R. Periaxin mutations causes recessive Dejerine-Sottas Neuropathy. *Am. J. Hum. Genet.* **2001**, *68*, 325–333.
151. Leal, A.; Morera, B.; Del Valle, G.; Heuss, D.; Kayser, C.; Berghoff, M.; Villegas, R.; Hernandez, E.; Mendez, M.; Hennies, H.C.; *et al.* A second locus for an axonal form of autosomal recessive Charcot-Marie-Tooth disease maps to chromosome 19q13.3. *Am. J. Hum. Genet.* **2000**, *68*, 269–274.
152. Leal, A.; Huehne, K.; Bauer, F.; Sticht, H.; Berger, P.; Suter, U.; Morera, B.; Del, V.G.; Lupski, J.R.; Ekici, A.; *et al.* Identification of the variant Ala335Val of MED25 as responsible for CMT2B2: molecular data, functional studies of the SH3 recognition motif and correlation between wild-type MED25 and PMP22 RNA levels in CMT1A animal models. *Neurogenetics* **2009**, *10*, 275–287.
153. Nishimura, A.L.; Mitne-Neto, M.; Silva, H.C.; Richieri-Costa, A.; Middleton, S.; Cascio, D.; Kok, F.; Oliveira, J.R.; Gillingwater, T.; Webb, J.; *et al.* A mutation in the vesicle-trafficking protein VAPB causes late-onset spinal muscular atrophy and amyotrophic lateral sclerosis. *Am. J. Hum. Genet.* **2004**, *75*, 822–831.
154. Inoue, K.; Shilo, K.; Boerkoel, C.F.; Crowe, C.; Sawady, J.; Lupski, J.R.; Agamanolis, D.P. Congenital hypomyelinating neuropathy, central dysmyelination, and Waardenburg-Hirschsprung disease: Phenotypes linked by SOX10 mutation. *Ann. Neurol.* **2002**, *52*, 836–842.
155. Nakhro, K.; Park, J.M.; Hong, Y.B.; Park, J.H.; Nam, S.H.; Yoon, B.R.; Yoo, J.H.; Koo, H.; Jung, S.C.; Kim, H.L.; *et al.* SET binding factor 1 (SBF1) mutation causes Charcot-Marie-Tooth disease type 4B3. *Neurology* **2013**, *81*, 165–173.
156. Kennerson, M.L.; Yiu, E.M.; Chuang, D.T.; Kidambi, A.; Tso, S.C.; Ly, C.; Chaudhry, R.; Drew, A.P.; Rance, G.; Delatycki, M.B.; *et al.* A new locus for X-linked dominant Charcot-Marie-Tooth disease (CMTX6) is caused by mutations in the pyruvate dehydrogenase kinase isoenzyme 3 (PDK3) gene. *Hum. Mol. Genet.* **2013**, *22*, 1404–1416.
157. Gal, A.; Mücke, J.; Theile, H.; Wieacker, P.F.; Ropers, H.H.; Wienker, T.F. X-linked dominant Charcot-Marie-Tooth disease: Suggestion of linkage with a cloned DNA sequence from the proximal Xq. *Hum. Genet.* **1985**, *70*, 38–42.
158. Fischbeck, K.H.; ar-Rushdi, N.; Pericak-Vance, M.; Rozear, M.; Roses, A.D.; Fryns, J.P. X-linked neuropathy: Gene localization with DNA probes. *Ann. Neurol.* **1986**, *20*, 527–532.

159. Bergoffen, J.; Scherer, S.S.; Wang, S.; Oronzi Scott, M.; Bone, L.J.; Paul, D.L.; Chen, K.; Lensch, M.W.; Chance, P.F.; Fischbeck, K.H. Connexin mutations in X-linked Charcot-Marie-Tooth disease. *Science* **1993**, *262*, 2039–2042.
160. Bergoffen, J.; Trofatter, J.; Pericak-Vance, M.A.; Haines, J.L.; Chance, P.F.; Fischbeck, K.H. Linkage localization of X-linked Charcot-Marie-Tooth disease. *Am. J. Hum. Genet.* **1993**, *52*, 312–318.
161. Kennerson, M.L.; Nicholson, G.A.; Kaler, S.G.; Kowalski, B.; Mercer, J.F.; Tang, J.; Llanos, R.M.; Chu, S.; Takata, R.I.; Speck-Martins, C.E.; *et al.* Missense mutations in the copper transporter gene ATP7A cause X-linked distal hereditary motor neuropathy. *Am. J. Hum. Genet.* **2010**, *86*, 343–352.
162. Takata, R.I.; Speck, M.; Passosbueno, M.; Abe, K.; Nishimura, A.; Dorvalina, D.; Monteiro, A.; Lima, M.; Kok, F.; Zatz, M. A new locus for recessive distal spinal muscular atrophy at Xq13.1-q21. *J. Med. Genet.* **2004**, *41*, 224–229.
163. Kim, H.J.; Sohn, K.M.; Shy, M.E.; Krajewski, K.M.; Hwang, M.; Park, J.H.; Jang, S.Y.; Won, H.H.; Choi, B.O.; Hong, S.H.; *et al.* Mutations in PRPS1, which encodes the phosphoribosyl pyrophosphate synthetase enzyme critical for nucleotide biosynthesis, cause hereditary peripheral neuropathy with hearing loss and optic neuropathy (CMTX5). *Am. J. Hum. Genet.* **2007**, *81*, 552–558.
164. Kim, H.J.; Hong, S.H.; Ki, C.S.; Kim, B.J.; Shim, J.S.; Cho, S.H.; Park, J.H.; Kim, J.W. A novel locus for X-linked recessive CMT with deafness and optic neuropathy maps to Xq21.32-q24. *Neurology* **2005**, *64*, 1964–1967.
165. Ionasescu, V.V.; Trofatter, J.; Haines, J.L.; Summers, A.M.; Ionasescu, R.; Searby, C. Heterogeneity in X-linked recessive Charcot-Marie-Tooth neuropathy. *Am. J. Hum. Genet.* **1991**, *48*, 1075–1083.
166. Priest, J.M.; Fischbeck, K.H.; Nouri, N.; Keats, B.J. A locus for axonal motor-sensory neuropathy with deafness and mental retardation maps to Xq24-q26. *Genomics* **1995**, *29*, 409–412.
167. Rinaldi, C.; Grunseich, C.; Sevrioukova, I.F.; Schindler, A.; Horkayne-Szakaly, I.; Lamperti, C.; Landoure, G.; Kennerson, M.; Burnett, B.G.; Bönnemann, C.; *et al.* Cowchock syndrome is associated with a mutation in apoptosis-inducing factor. *Am. J. Hum. Genet.* **2012**, *91*, 1095–1102.
168. Huttner, I.G.; Kennerson, M.L.; Reddel, S.W.; Radovanovic, D.; Nicholson, G.A. Proof of genetic heterogeneity in X-linked Charcot-Marie-Tooth disease. *Neurology* **2006**, *67*, 2016–2021.
169. Brewer, M.; Changi, F.; Antonellis, A.; Fischbeck, K.; Polly, P.; Nicholson, G.; Kennerson, M. Evidence of a founder haplotype refines the X-linked Charcot-Marie-Tooth (CMTX3) locus to a 2.5 Mb region. *Neurogenetics* **2008**, *9*, 191–195.
170. Pitceathly, R.D.; Murphy, S.M.; Cottenie, E.; Chalasani, A.; Sweeney, M.G.; Woodward, C.; Mudanohwo, E.E.; Hargreaves, I.; Heales, S.; Land, J.; *et al.* Genetic dysfunction of MT-ATP6 causes axonal Charcot-Marie-Tooth disease. *Neurology* **2012**, *79*, 1145–1154.

© 2014 by the authors; licensee MDPI, Basel, Switzerland. This article is an open access article distributed under the terms and conditions of the Creative Commons Attribution license (http://creativecommons.org/licenses/by/3.0/).
